# Versatile generation of precise gene edits in bovines using SEGCPN

**DOI:** 10.1186/s12915-023-01677-0

**Published:** 2023-10-20

**Authors:** Ming Wang, Fangrong Ding, Haiping Wang, Ling Li, Yunping Dai, ZhaoLin Sun, Ning Li

**Affiliations:** 1 College of Animal Science and Technology, China Agricultural University, No. 2 Yuanmingyuan Xilu, Beijing, 100193 China; 2 College of Biological Sciences, China Agricultural University, No. 2 Yuanmingyuan Xilu, Beijing, 100193 China; 3 Beijing Capital Agribusiness Future Biotechnology Co., Ltd, No. 75 Bingjiaokou Hutong, Beijing, 100088 China

**Keywords:** Bovine, Precise gene editing, Point mutation, Targeted deletion, Gene tagging, Gene replacement

## Abstract

**Background:**

Gene knockout and knock-in have been widely performed in large farm animals based on genome editing systems. However, many types of precise gene editing, including targeted deletion, gene tagging, and large gene fragment replacement, remain a challenge in large farm animals.

**Results:**

Here, we established versatile self-excising gene-targeting technology in combination with programmable nucleases (SEGCPN) to efficiently generate various types of precise gene editing in bovine. First, we used this versatile method to successfully generate bovine embryos with point mutations and 11-bp deletions at the *MSTN* locus. Second, we successfully generated bulls with EGFP labeling at the *SRY* locus. Finally, we successfully generated humanized cows in which the endogenous 18-kb α-casein gene was replaced with a 2.6-kb human α-lactalbumin gene.

**Conclusions:**

In summary, our new SEGCPN method offers unlimited possibilities for various types of precise gene editing in large animals for application both in agriculture and disease models.

**Supplementary Information:**

The online version contains supplementary material available at 10.1186/s12915-023-01677-0.

## Background

The ability to perform virtually any type of precise gene editing, such as point mutation, targeted deletion, gene tagging, and large gene fragment replacement, in the genome of large farm animals is very important for the application of human disease models and improving agricultural traits. Despite rapid advances in genome editing technologies, the majority of economic trait-associated genetic variants in large farm animals [1] remain difficult to mimic and change.

Currently, genetic engineering in large farm animals, such as the generation of gene knockouts or knock-ins and various chromosomal rearrangements, involves the use of programmable nucleases, such as TALENs and CRISPR/Cas9 systems [2], which are widely used in a variety of cell lines and organisms [3, 4]. Programmable nucleases make double-strand DNA breaks (DSBs) that can disrupt genes by inducing mixtures of insertions and deletions (indels) at target sites. However, DSBs are difficult to introduce targeted insertions or deletions [5]. Homology-directed repair (HDR) stimulated by DSBs has been widely used for precise gene editing. However, HDR relies on exogenous donor DNA repair templates, typically including the selectable markers [6, 7] that confound functional studies [8, 9].

Point mutation has generally been achieved by the cointroduction of programmable nucleases and single-stranded oligonucleotides in cows [10], pigs [11, 12], sheep [13], and goats [14]. However, this technique has disadvantages, such as low efficiency, mosaic, and random integration. Additionally, newly developed base editors (BEs) have been used to precisely edit target bases (C.G-to-T.A or A.T-to-G.A, conversion) in pigs [15–17], goats [18], and sheep [19]. However, this technique still cannot produce arbitrary nucleotide mutations at any position and has yet to be reported in bovine. Recently, a prime editing (PE) system that enables the generation of targeted insertions, deletions, and all 12 classes of point mutations without requiring DSBs or a DNA donor repair template was shown to function efficiently in mammalian cells [5], mice [20], and plants [21]. However, it is difficult to complete the insertion and replacement of long fragments larger than 50 bp. Moreover, this technology has not yet been reported in any large animal research.

In addition, research involving gene replacement technology has been established in pigs and cattle, allowing the generation of humanized HD model pigs [22] and hornless dairy cattle [23], by the cointroduction of programmable nucleases and double-strand donor. However, in order to achieve precise gene replacement and avoid the influence of selective marker on later phenotypes, the marker gene was not introduced in the donor vector in these two studies, so it needed to screen a large number of cell clones, and the positive rate was very low, 0.37% (9/2430) and 2% (5/226), respectively. Moreover, these technologies only allow the replacement of small gene fragments up to 600 bp. To our knowledge, targeted deletion, gene tagging, and large gene fragment replacement technology have not been reported in large farm animals.

Here, we describe the development of a versatile genome editing method that mediates point mutation, targeted deletion, gene tagging, and large gene fragment replacement, which we have named self-excising gene-targeting technology in combination with programmable nucleases (SEGCPN). Our technology consists of two components: a new self-excision gene-targeting system and programmable nuclease, such as a TALEN or Cas9. The self-excision element consisting of the embryo-specific truncated m*Oct4* promoter (TmO2.5) driving the expression of *Cre*, the selectable marker *neo,* and two *loxP* sites, and this element was substituted into the positive selection marker of a traditional gene-targeting vector to obtain the self-excision gene-targeting system. We then tested the efficacy of SEGCPN for introducing various types of precise gene editing. We used SEGCPN to successfully generate bovine embryos with point mutations and 11-bp deletions at the *MSTN* locus, bulls with EGFP labeling at the *SRY* locus, and gene replacement humanized cows. This technology has valuable potential to achieve various gene editing in large farm animals for biomedical and agricultural applications.

## Results

### Establishment of SEGCPN

**Fig. 1:**
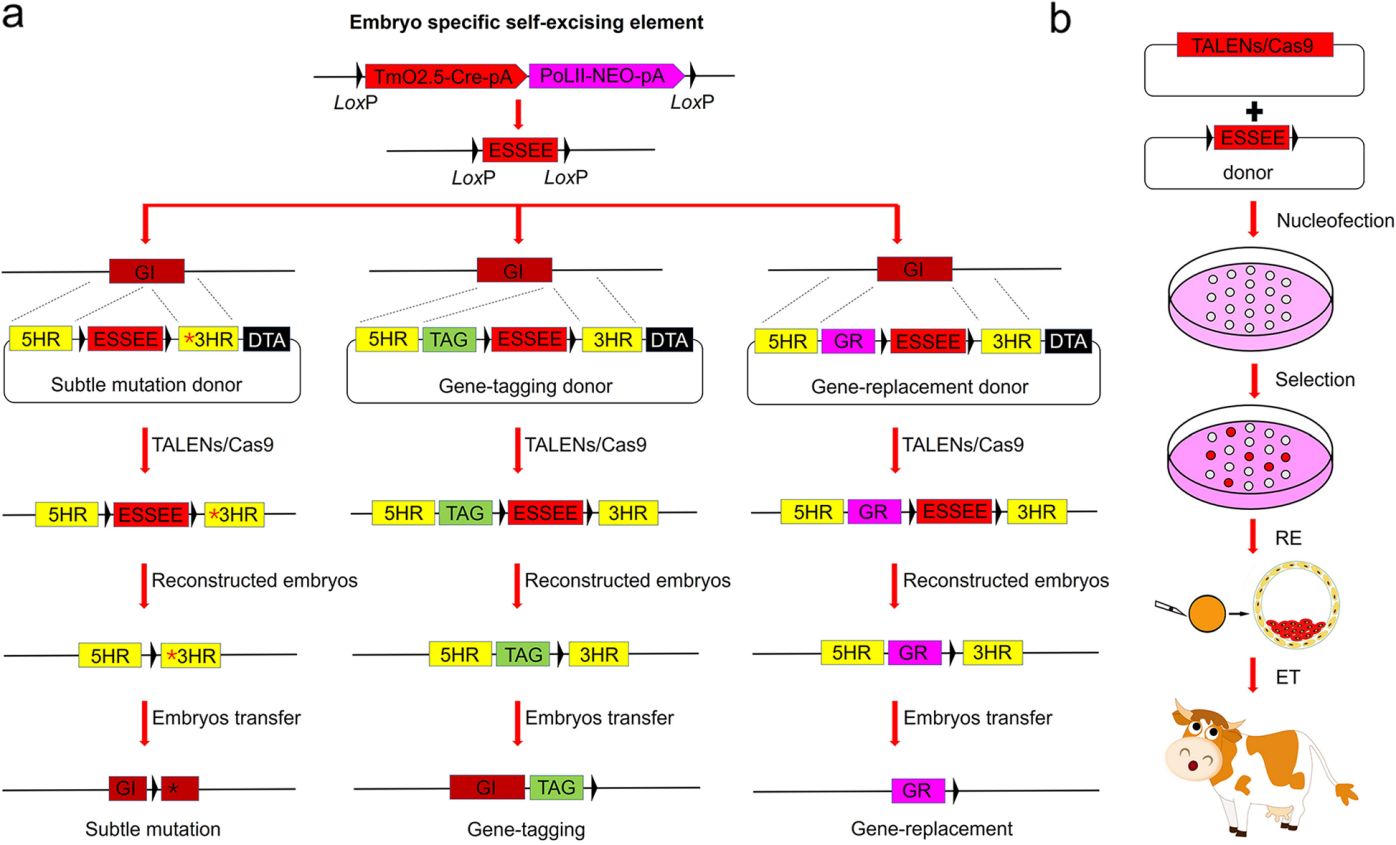
Scheme for performing various types of precise gene editing based on embryo-specific self-excising gene targeting. **a** Scheme for performing various types of precise gene editing. The embryo-specific self-excising element (ESSEE) was cloned into the gene-targeting vector. BFFs identified to have modifications at the specific locus were used for SCNT to generate genetically modified blastocysts or individual animals. GI, gene of interest for precise gene editing; *, subtle mutation; TAG, gene-tagging gene; GR, gene-replacement gene; DTA, diphtheria toxin A as negative selection marker. **b** Scheme of nucleofection, selection, embryo reconstruction, and embryo transferation using the SEGCPN

There is a lack of broadly applicable and rapid methods for achieving precise gene editing in farm animals. To overcome this hurdle, we developed a new method, named SEGCPN, for achieving various types of precise gene editing in bovine (Fig. 1). First, we devised an embryo-specific self-excision element (ESSEE) by taking advantage of an embryo-specific promoter to drive the expression of *Cre* recombinase. The positive selection marker gene linked to *Cre* survived selection in cultured cells but was self-removed as it passed through the stages of embryonic development. Second, different ESSEE gene-targeting donor vectors were constructed based on the different types of precise gene editing desired, such as point mutation, targeted deletion, gene tagging, or large gene fragment replacement. Third, we cotransfected the ESSEE gene-targeting donor and programmable nucleases into bovine fetal fibroblasts (BFFs) and then obtained positive knock-in clones through positive and negative screening. Finally, we identified embryos and animals with positive precise gene editing in the F0 generation by traditional somatic cell nuclear transfer (SCNT) technology. As a short promoter facilitates the manipulation of the constructed vector, we cloned the 2.5-kb sequence upstream of m*Oct4*, containing four core regions (CR1 to CR4), to obtain a truncated m*Oct4* promoter (TmO2.5) for use in our subsequent experiments. To confirm that the TmO2.5 promoter could initiate gene expression in bovine preimplantation embryos, we constructed a pTmO2.5-EGFP vector in which the EGFP reporter gene was driven by TmO2.5 (Additional file 1: Fig. S1a). The linearized pTmO2.5-EGFP vector was electroporated into BFFs, and positive colonies were identified by polymerase chain reaction (PCR) analysis using the primers EGFP-F and EGFP-R (Additional file 1: Fig. S1a); positive colonies were then used as donor cells for SCNT. Fluorescence imaging of the resulting embryos indicated that EGFP was expressed from the four-cell stage to the blastocyst stage under the control of TmO2.5, similar to the EGFP expression pattern observed in mice and cows driven by the 18-kb *mOct4* promoter, while there was no expression at the positive cell colonies (Additional file 1: Fig. S1b). Thus, TmO2.5 was concluded to be an ideal embryo-specific promoter in this study.

To further determine whether TmO2.5 can drive the recombination functions of *Cre* in bovine embryos, we constructed a TmO2.5-Cre reporter vector (pTmOC-S-Cre) containing the *CAG* promoter followed by a STOP cassette flanked with two *loxP*s, the *EGFP* gene, TmO2.5, and the *Cre* gene and stably transfected it into BFFs. Positive colonies were identified by PCR analysis using the primers mOCT4-F and mOCT4-R (Additional file 1: Fig. S1c) and were used as donor cells for SCNT. Fluorescence imaging of the positive colonies showed no EGFP expression, while the resulting embryos showed ubiquitously EGFP expression from the eight-cell stage to the blastocyst stage (Additional file 1: Fig. S1d), indicating that TmO2.5 drove expression of *Cre*, which successfully excised the STOP cassette to allow *EGFP* transcription. Thus, TmO2.5-Cre can specifically excise genes of interest in bovine embryos. According to these results, we further constructed the embryo-specific self-excision element (ESSEE), it consists of three parts: first, TmO2.5 driving *Cre*; second, the POLII promoter driving the marker gene *Neo*; and third, two *loxP* sites with the same direction were located at either end. The target vector containing the ESSEE was transfected into BFFs, because the TmO2.5 could not drive the expression of *Cre* in cells, while the marker gene *Neo* could be activated by POLII promoter, normal cell screening could be performed and the positive cells were enriched. After obtaining the positive clonies, during the process of embryo reconstruction and in the early embryo development in vitro, TmO2.5 drove the expression of *Cre* and ESSEE achieved self-excision. We believed the ESSEE was applicable and could be used for further research.

#### Generation of blastocysts and fetuses with MSTN point mutation using SEGCPN

**Fig. 2:**
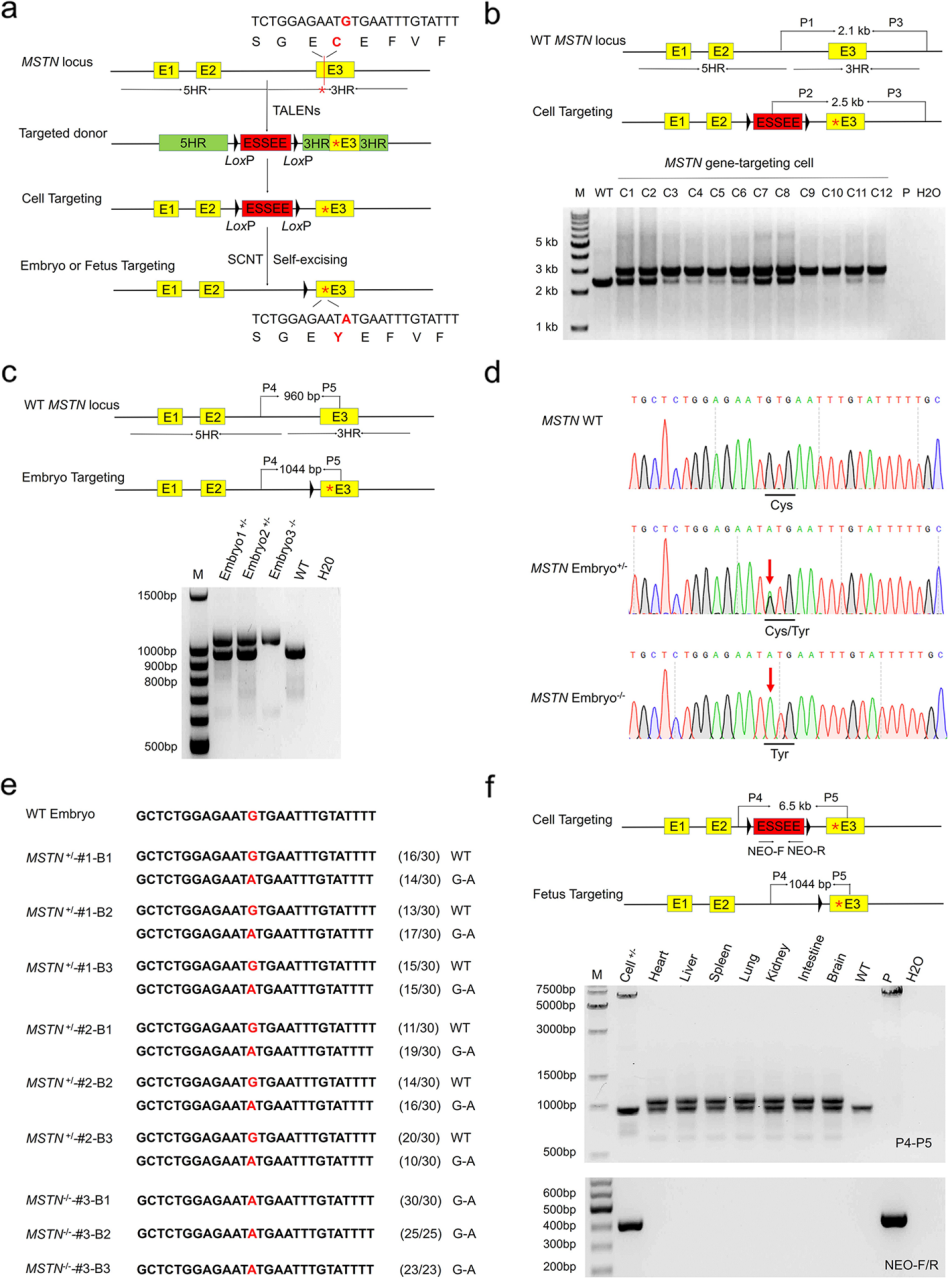
Generation of blastocysts and fetuses with *MSTN* point mutations using SEGCPN. **a** Procedure for generating blastocysts and fetuses with *MSTN* point mutations using SEGCPN. **b** Identification of *MSTN* point mutation-edited cell clones by PCR. M, 1-kb DNA ladder; C1–C12, *MSTN*-edited clones; P, donor vector; WT, wild-type cells; H_2_O, negative control. **c** Identification of *MSTN* point mutation-edited blastocysts by PCR. M, 100-bp DNA ladder; embryo^+/−^, heterozygous *MSTN*-edited embryos; embryo^−/−^, homozygous *MSTN*-edited embryos; WT, wild-type embryos; H_2_O, negative control. **d** Sanger sequencing chromatograms of DNA from WT and *MSTN* mutant blastocysts. The red arrow indicates the substituted nucleotide. Relevant codon identities at the target site are presented beneath the DNA sequence.** e** Alignments of mutant sequences from the TA-cloning sequencing of a single embryo. The G.C-to-A.T point mutation site is shown in red. The column on the right indicates the frequencies of mutant alleles. WT, wild-type embryos. **f** Identification of mosaicism in a heterozygous *MSTN* point mutation-edited fetus. P4/P5, identification of *MSTN*^+/−^ gene editing in various fetal tissues. Neo-F/R, the marker gene in the ESSEE detected in various fetal tissues. Cell^+/−^, heterozygous positive gene-edited clone; lanes 3–9, various tissues of an *MSTN*^+/−^-edited fetus; WT, wild-type tissue; P, donor vector; H_2_O, negative control

*MSTN* is a member of the transforming growth factor beta (TGF-β) superfamily, which acts as a negative regulator of muscle growth. Piedmontese cattle are a well-known double-muscled breed whose *MSTN* gene contains a missense mutation in exon 3, resulting in the substitution of tyrosine for an invariant cysteine in the mature region of the protein [24]. To validate the SEGCPN method, we attempted to simulate this natural mutation to generate a G.C-to-A.T point mutation in *MSTN*. The procedure for generating the *MSTN* G.C-to-A.T point mutation in bovine is shown in Fig. 2a. We engineered three pairs of TALENs directed against bovine *MSTN* intron 2 (Additional file 1: Fig. S2a) and used T7 endonuclease I (T7EI) assays to test whether these TALENs could successfully edit the *MSTN* gene in BFFs. TALEN pairs M1 and M2 showed gene modification efficiencies of 25% and 11%, respectively, and the modifications were subsequently verified by TA cloning and sequencing (Additional file 1: Fig. S2b, c). As TALENs-M1 cleaved the target site with greater efficiency, we used TALENs-M1 in subsequent experiments.

TALENs-M1 and the gene-targeting vector pMSTN-ESSEE (point mutation) were co-electroporated into BFFs, and positive clones were selected with G418. As shown in Additional file 2: Table S1, we screened 48 single-cell clones and obtained 31 targeted clones, including 2 homozygous targeted clones, by PCR genotyping that could distinguish the targeted *MSTN* and WT alleles. PCR analysis with the P1 and P3 primers showed the expected 2.1-kb band for the WT allele, while analysis with the P2 and P3 primers showed the expected 2.5-kb band for the targeted *MSTN* allele (Fig. 2b). Sequencing further confirmed that correct targeting was achieved (Additional file 1: Fig. S3a).

Subsequently, 2 heterozygous targeted clones and 1 homozygous targeted clone were used as donors for nuclear transfer (Additional file 2: Table S2). Day 7 blastocysts derived from these three clones were collected to examine the G.C-to-A.T point mutation and the presence of ESSEE including the marker gene. As expected, the edited *MSTN* allele produced a 1044-bp band, and the WT allele produced a 960-bp band when analyzed with the primers P4 and P5 (Fig. 2c). PCR sequencing also confirmed the successful generation of the *MSTN* G.C-to-A.T point mutation (Fig. 2d). TA cloning sequencing showed that single embryos derived from the three clones were homozygous or heterozygous for the G.C-to-A.T point mutation at the expected site (Fig. 2e).

Moreover, to determine whether there is the existence of any mosaicism, one heterozygous fetus was generated, and the excision of the ESSEE was detected in various tissues. PCR assays and DNA sequencing demonstrated the absence of mosaicism in the fetus (Fig. 2f; Additional file 1: Fig. S3b). To test for the potential non-specific mutations induced by the introduction of the TALENs, 4 potential off-target loci of TALENs-M1 were predicted (Additional file 2: Table S3). T7EI analysis was used to determine the off-target effects. The results revealed that no off-target effects occurred in this heterozygous fetus (Additional file 1: Fig. S4a). These results suggested that point mutations can be obtained using the SEGCPN method.

### Generation of blastocysts and fetuses with an 11-bp MSTN deletion mutation using SEGCPN

**Fig. 3:**
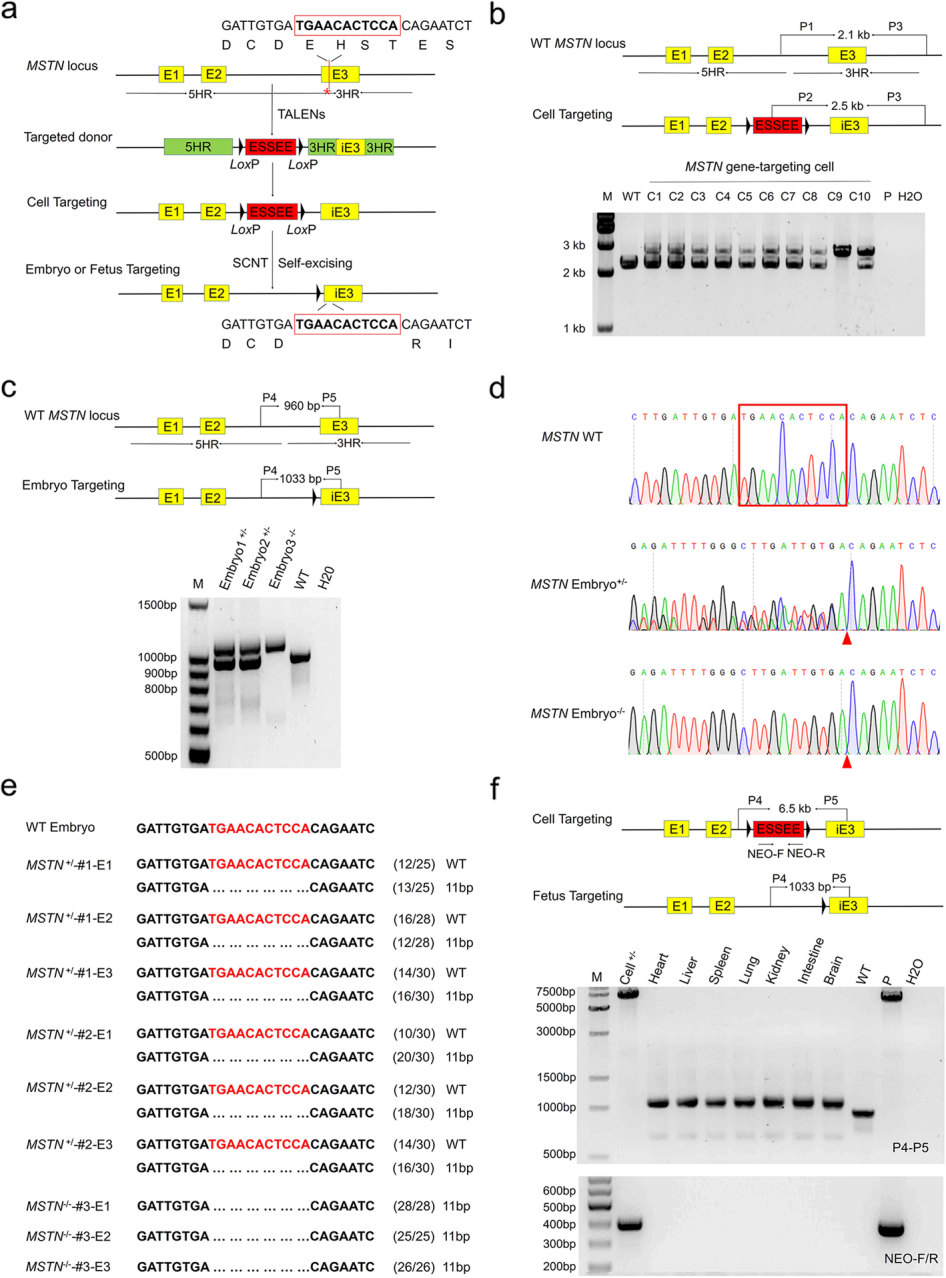
Generation of blastocysts and fetuses with an 11-bp *MSTN* deletion using SEGCPN. **a** Procedure for generating blastocysts and fetuses with an 11-bp *MSTN* deletion using SEGCPN. **b** Identification of *MSTN* 11-bp deletion edited cell clones by PCR. M, 1-kb DNA ladder; C1–C10, *MSTN*-edited clones; P, donor vector; WT, wild-type cells; H_2_O, negative control. **c** Identification of *MSTN* 11-bp deletion-edited blastocysts by PCR. M, 100-bp DNA ladder; embryo^+/−^, heterozygous *MSTN*-edited embryos; embryo^−/−^, homozygous *MSTN*-edited embryos; WT, wild-type embryos; H_2_O, negative control. **d** Sanger sequencing chromatograms of DNA from WT and *MSTN* mutant blastocysts. The red arrowhead indicates the 11-bp deletion. **e** Alignments of mutant sequences from the TA-cloning sequencing of a single embryo. The 11-bp deletion site is shown in red. The column on the right indicates the frequencies of mutant alleles. WT, wild-type embryos. **f** Identification of mosaicism in a homozygous *MSTN*^−/−^ 11-bp deletion edited fetus. P4/P5, identification of *MSTN*^−/−^ gene editing in various fetal tissues. Neo-F/R, the marker gene in the ESSEE detected in various fetal tissues. Cell^−/−^, homozygous positive gene-edited clone; lanes 3–9, various tissues of the *MSTN*^−/−^ fetus; WT, wild-type tissue; P, donor vector; H_2_O, negative control

Belgian blue cattle are another well-known double-muscled breed whose *MSTN* gene contains an 11-bp deletion in exon 3, resulting in a frameshift mutation in the mature region of the protein [24, 25]. To validate the SEGCPN method, we attempted to simulate the natural mutation to generate an 11-bp *MSTN* deletion. The procedure for generating the 11-bp *MSTN* deletion in bovine is shown in Fig. 3a.

TALENs-M1 and the gene-targeting vector pMSTN-ESSEE (deletion) were co-electroporated into BFFs, and positive clones were selected with G418. As shown in Additional file 2: Table S4, we screened 44 single-cell clones and obtained 36 targeted clones, including 1 homozygous targeted clone, by PCR genotyping that could distinguish the targeted *MSTN* and WT alleles. PCR analysis with the P1 and P3 primers showed the expected 2.1-kb band for the WT allele, while with the P2 and P3 primers showed the expected 2.5-kb band for the targeted *MSTN* allele (Fig. 3b). Sequencing further confirmed that the correct targeting was achieved (Additional file 1: Fig. S5a).

Subsequently, 2 heterozygous targeted clones and 1 homozygous targeted clone were used as donors for nuclear transfer (Additional file 2: Table S5). Day 7 blastocysts derived from these three clones were collected to examine the 11-bp deletion and the presence of the marker gene. As expected, the edited *MSTN* allele produced a 1033-bp band, and the WT allele produced a 960-bp band when amplified with the primers P4 and P5 (Fig. 3c). PCR sequencing also confirmed the successful generation of the 11-bp *MSTN* deletion (Fig. 3d). TA cloning sequencing showed that single embryos derived from the three clones were homozygous or heterozygous for 11-bp deletion at the expected site (Fig. 3e).

Moreover, to determine the existence of any mosaicism, one homozygous fetus was generated, and the excision of the ESSEE was detected in various tissues. PCR assays and DNA sequencing showed the absence of mosaicism in the fetus (Fig. 3f; Additional file 1: Fig. S5b). HE staining was further used to determine the phenotype of the homozygous 11-bp deletion fetus, the result showed that the average size of muscle fibers from a fetus with *MSTN* homozygous 11-bp deletion (1812.80 ± 474.54 μm^2^) was substantially larger than that of the wild-type fetus of the same age (1097.00 ± 183.82 μm^2^), and the diameter of myofibers in the *MSTN*^−/−^ fetus (48.04 ± 7.92 μm) was also significantly larger than that in the control fetus (37.37 ± 3.94 μm) (Additional file 1: Fig. S6). Finally, the potential non-specific mutations induced by the introduction of the TALENs-M1 in this fetus were also detected. Sanger sequencing and T7EI analysis revealed that no off-target effects occurred in the homozygous 11-bp deletion fetus (Additional file 2: Table S3; Additional file 1: Fig. S4b,c). These results suggested that arbitrary nucleotide mutations at any position can be obtained using the SEGCPN method.

### Generation of bulls with an EGFP-labeled SRY gene on the Y chromosome using SEGCPN

**Fig. 4:**
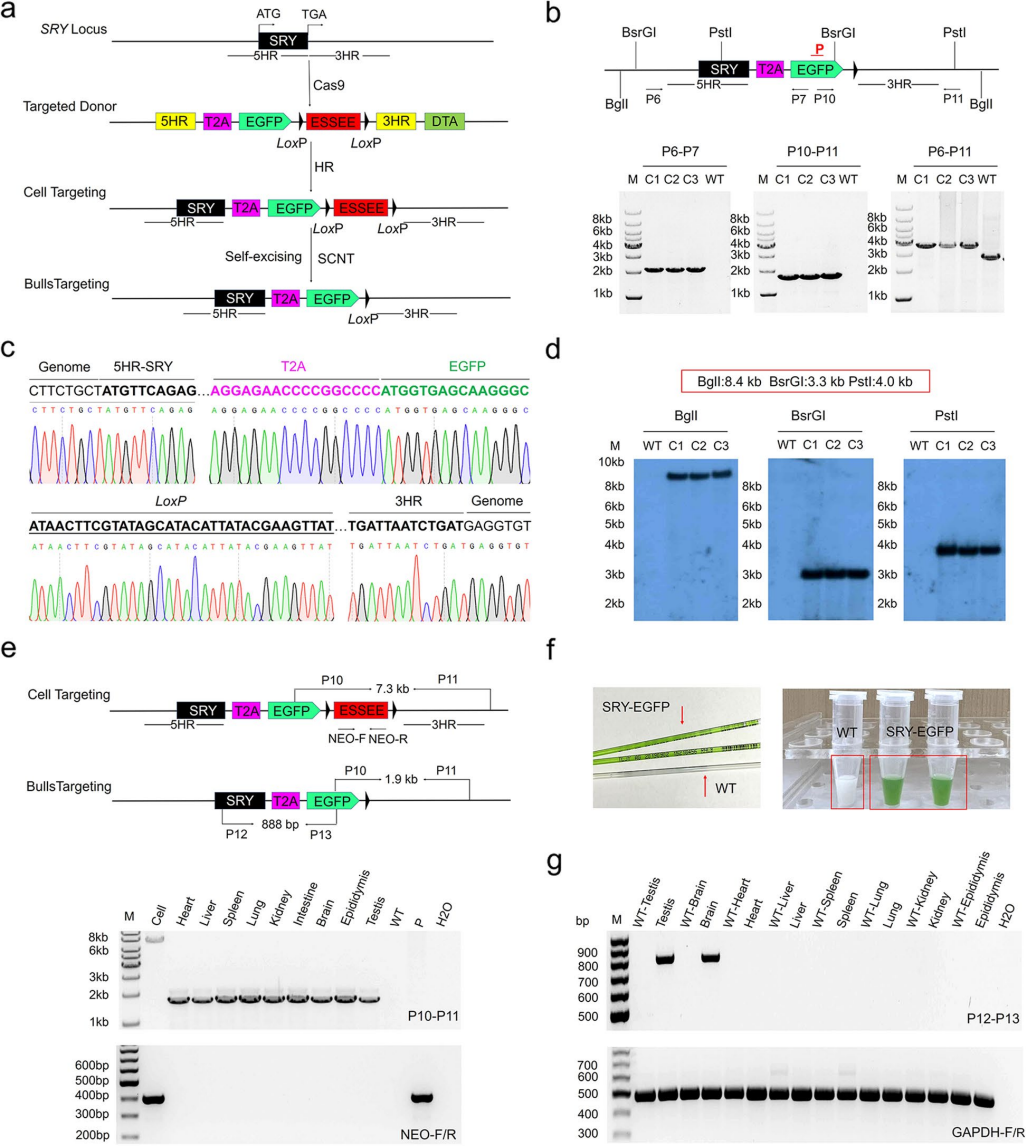
Generation of bulls with EGFP labeling at the *SRY* locus using SEGCPN. **a** Procedure for generating bulls with EGFP-labeling at the *SRY* locus using SEGCPN. **b** Schematic representation of the PCR analysis of the EGFP-labeled bovine. Primers P6 and P7 amplified a 2.2-kb product, primers P10 and P11 amplified a 1.9-kb product, and primers P6 and P11 amplified a 3.8-kb product to confirm positive EGFP-labeled bulls. M, 1-kb DNA ladder; C1–C3, EGFP-labeled bulls; WT, wild-type bull. **c** DNA sequencing between endogenous and exogenous DNA corresponding to homologous recombination in the EGFP-labeled bulls. **d** Southern blotting analysis of the EGFP-labeled bulls. The red rectangular box represents the positive band size obtained using different enzymes. Upon digestion with BglII, a band of 8.4 kb resulting from the targeted introduction of pSRY-EGFP was detected; using BsrGI, the expected fragment size was 3.3 kb; using PstI, the expected fragment size was 4.0 kb. C1–C3, EGFP-labeled bulls; WT, wild-type bull. **e** Identification of mosaicism in an EGFP-labeled bull. P10/P11, identification of precise EGFP-labeling in various bull tissues. Neo-F/R, the marker gene detected in various bull tissues. Cell, positive gene-targeted clone; lanes 3–11, various tissues of an EGFP-labeled bull; WT, wild-type tissue; P, donor vector; H_2_O, negative control. **f** Photos of semen from the EGFP-labeled bulls and WT bulls. **g** RT-PCR analysis of EGFP expression in tissues from EGFP-labeled bulls. GAPDH was used as a reference

The bovine *SRY* gene is reported to be a single-copy gene on the Y chromosome [26] and served as the main genetic switch for male sex development in bovine [27], and the *SRY* gene is also expressed in bovine sperm [28]. To validate that the SEGCPN method we built was a simple tool, we attempted to label the *SRY* gene to generate EGFP-labeled bulls to establish a simpler, better transgene-based system for sexing control [29]. The procedure for generating EGFP-labeled bulls is shown in Fig. 4a. We designed three single-guide RNA (sgRNA) sequences to target the *SRY* gene (Additional file 1: Fig. S7a). The three sgRNA sequences were separately cloned into the pX330 plasmid to yield pX330-sgRNA1, pX330-sgRNA2, and pX330-sgRNA3. T7E1 assay was used to assess the frequencies of Cas9-induced indels in BFFs. pX330-sgRNA1, pX330-sgRNA2, and pX330-sgRNA3 showed gene modification efficiencies of 42%, 20%, and 12%, respectively (Additional file 1: Fig. S7b), and the modifications were subsequently verified by TA cloning and sequencing (Additional file 1: Fig. S7c). As pX330-sgRNA1 cleaved the target site with the greatest efficiency, we used pX330-sgRNA1 in subsequent experiments.

pX330-sgRNA1 and the gene-targeting vector pSRY-ESSEE-EGFP were co-electroporated into BFFs, and positive clones were selected with G418. As shown in Additional file 2: Table S6, we screened 41 single-cell clones and obtained 28 correctly targeted clones by PCR genotyping. Primers P6 and P7 amplified the expected 2.1-kb band, and primers P8 and P9 amplified the expected 1.9-kb band (Additional file 1: Fig. S8a). The PCR products were also identified by sequencing (Additional file 1: Fig. S8b, c).

Subsequently, the positive clones were used as donors for nuclear transfer. In total, we transferred 160 transgenic cloned blastocysts into 80 recipients, and a total of three cloned calves were born alive (Additional file 2: Table S7). The calves were subjected to the following detection strategy. With the P6/P7 primer set, located between the donor and outside of the 5′ homologous arm, the expected 2.2-kb band was amplified; with the P10/P11 primer set, located between the donor and outside of the 3′ homologous arm, the expected 1.9-kb band was amplified; and with the P6/P11 primer set for the sequences flanking the 5′ and 3′ homologous arms, the expected 3.8-kb band was amplified. These results confirmed that the EGFP had been knocked into the *SRY* gene, and the ESSEE including the marker gene was removed successfully (Fig. 4b). Similar findings were obtained by DNA sequencing (Fig. 4c) and Southern blotting (Fig. 4d). We also detected the excision of the marker in various calf tissues through PCR (Fig. 4e) and found no mosaicism in the calves. Then, off-target effects of the pX330-sgRNA1 were also evaluated, 4 potential off-target loci of pX330-sgRNA1 were predicted (Additional file 2: Table S8). Sanger sequencing and T7EI analysis were used to determine the off-target effects. The results revealed that no off-target effects occurred in these three cloned calves (Additional file 1: Fig. S9).

Semen from the EGFP-labeled bulls appeared green, while the WT semen was off-white or pale yellow (Fig. 4f). Because the *SRY* promoter drove EGFP expression, we tested whether its expression pattern recapitulated that of endogenous *SRY* expression. We performed semiquantitative RT-PCR to detect EGFP transcript levels in various tissues. The results confirmed that EGFP was expressed in the gonads and brain but not in other tissues (Fig. 4g), consistent with previous reports in mice. Immunohistochemical analysis further confirmed that the EGFP-labeled bulls’ genitals had normal histological structure and EGFP expression (Additional file 1: Fig. S10). These results suggested that the SEGCPN is a rapid and easy method for the generation of gene-tagged bovine.

### Generation of cows with site-specific gene replacement using SEGCPN

**Fig. 5:**
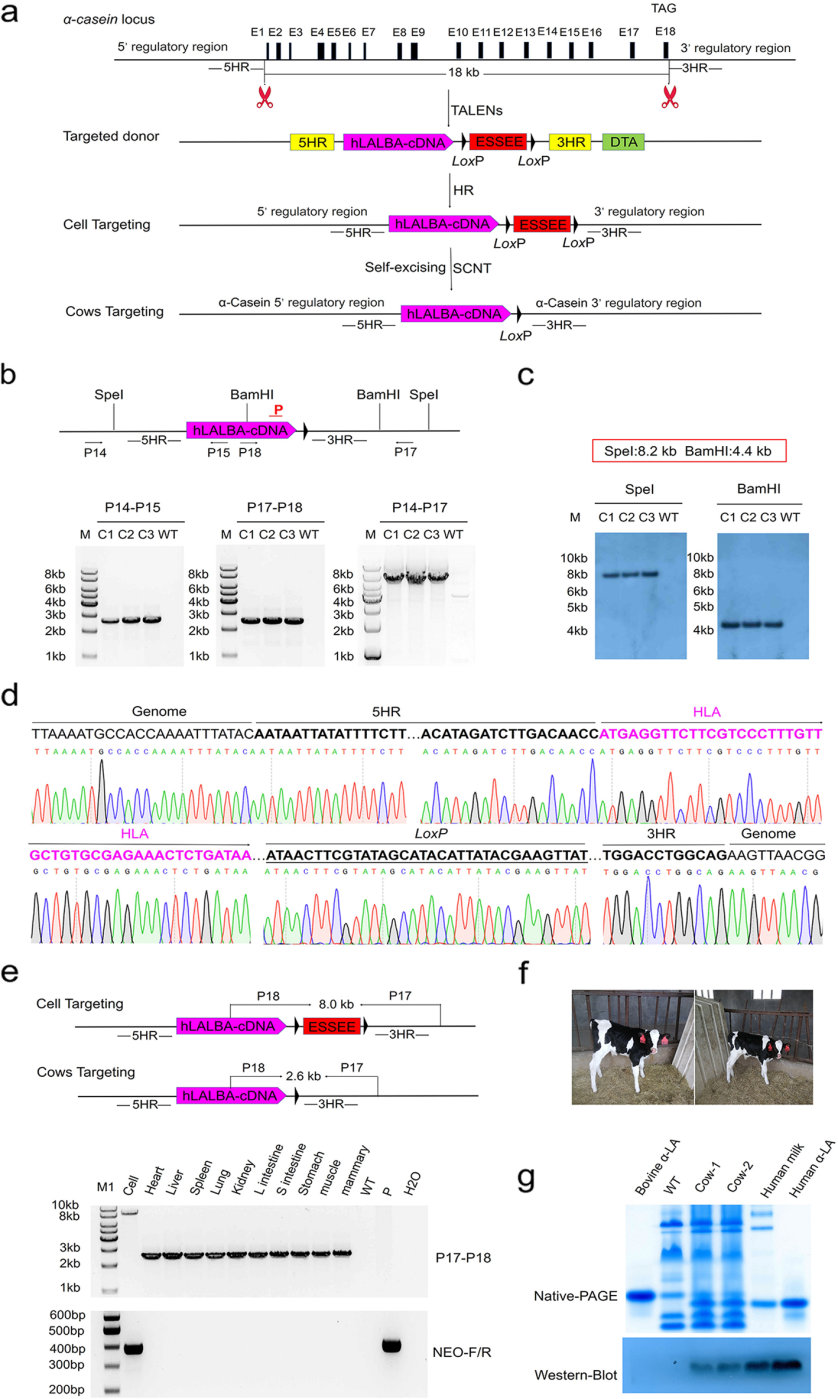
Generation of cows with site-specific gene replacement using SEGCPN. **a** Procedure for generating cows with site-specific gene replacement using SEGCPN. **b** Schematic representation of the PCR analysis of the cows with site-specific gene replacement. Primers P14 and P15 amplified a 2.7-kb product, primers P17 and P18 amplified a 2.6-kb product, and primers P14 and P17 amplified a 6.8-kb product to confirm positive gene replacement in cows. M, 1-kb DNA ladder; C1–C3, gene replacement cows; WT, wild-type cow. **c** Southern blotting analysis of the gene replacement cows. The red rectangular box represents the positive band size obtained using different enzymes. Using SpeI, the expected fragment size was 8.2 kb; using the BamHI, the expected fragment size was 4.4 kb. C1–C3, gene replacement cows; WT, wild-type cow. **d** DNA sequencing between endogenous and exogenous DNA corresponding to homologous recombination in the gene replacement cows. **e** Identification of mosaicism in a gene replacement cow. P17/P18, identification of precise gene replacement in various cow tissues. Neo-F/R, the marker gene detected in various cow tissues. Cell, positive gene-targeted clone; lanes 3–12, various tissues of the gene replacement cow; WT, wild-type tissue; P, donor vector; H_2_O, negative control. **f** Image of the gene replacement cows. **g** Analysis of the expression of recombinant human α-LA. Whey protein from the gene replacement cows and wild-type cows was separated by native PAGE and then subjected to blotting and hybridization with an anti-HLA antibody. Lane 1, bovine α-LA; lane 2, bovine whey; lanes 3–4, transgenic whey from the gene replacement cows; lane 5, human whey; lane 6, human α-LA

The ability to express human proteins in cow milk is desirable both for enhancing the nutritional value of cow milk and for supplying human proteins for pharmaceutical research or production. In this study, we attempted to establish a rapid and easy method for generating genetically humanized cows by replacing the endogenous 18-kb α-casein gene (*CSN1*) with a 2.6-kb *LALBA* gene to express the human alpha-lactalbumin (HLA) protein. The procedure for generating gene replacement cows is shown in Fig. 5a. We engineered three pairs of TALENs directed against bovine *CSN1* exon 1 and exon 18. T7EI assay was used to test the nuclease activity of these TALENs in BFFs. TALEN pairs C1, C2, and C3, directed against bovine *CSN1* exon 1, showed gene modification efficiencies of 0%, 24%, and 26%, respectively, and TALEN pairs C4, C5, and C6, directed against bovine *CSN1* exon 18, showed gene modification efficiencies of 18%, 1%, and 20%, respectively. The modifications were subsequently verified by TA cloning and sequencing (Additional file 1: Fig. S11, S12). TALENs-C3 and TALENs-C6 cleaved the target sites with the greatest efficiency, so we used these two pairs in subsequent experiments.

TALENs-C3, TALENs-C6, and the linearized gene-targeting vector pHLA-ESSEE-RE were co-electroporated into BFFs, and positive clones were selected with G418. As shown in Additional file 2: Table S9, we screened 98 single-cell clones and obtained 56 correctly targeted clones by PCR genotyping. Primers P14 and P15 amplified the expected 2.7-kb band, and primers P16 and P17 amplified the expected 2.7-kb band (Additional file 1: Fig. S13a). The PCR products were also identified by sequencing (Additional file 1:Fig. S13b, c).

Subsequently, the positive clones were used as donors for nuclear transfer. In total, we transferred 100 transgenic cloned blastocysts into 60 recipients, and a total of three heterozygous heifers were born (Additional file 2: Table S10). The heifers were subjected to the following detection strategy. With the P14/P15 primer set, located between the donor and outside of the 5′ homologous arm, the expected 2.7-kb band was amplified; with the P17/P18 primer set, located between the donor and outside of the 3′ homologous arm, the expected 2.6-kb band was amplified; and with the P14/P17 primer set for the sequences flanking the 5′ and 3′ homologous arms, the expected 6.8-kb band was amplified. These results confirmed that the endogenous *CSN1* had been replaced with the 2.6-kb *LALBA* gene, and the ESSEE including marker gene was removed successfully (Fig. 5b; Additional file 1: Fig. S14). Similar findings were obtained by Southern blotting and DNA sequencing (Fig. 5c, d). We also detected the excision of the ESSEE in various heifer tissues through PCR (Fig. 5e) and found no mosaicism in the heifers. The heifers exhibited normal growth and two of them remained alive (Fig. 5f). The potential non-specific mutations induced by the introduction of the TALENs-C3 and TALENs-C6 were predicted (Additional file 2: Table S11, S12). Sanger sequencing and T7EI analysis revealed that no off-target effects occurred in these three cloned heifers (Additional file 1: Fig. S15, S16).

To confirm that the cows with heterozygous gene replacement expressed HLA, milk from two cows was collected by prolactin treatment. Native-PAGE and Western blotting verified the desired gene replacement in cows expressing HLA (Fig. 5g). ELISA was further used to quantify the human HLA expression levels in the milk. As shown in Table 1, the HLA concentrations in milk from these two cows were 1.6 g/L and 1.5 g/L, respectively. We also analyzed the components of milk. The gross composition, including the lactose, total protein, total fat, and total solids of whole milk samples from the gene replacement cows had no significant difference compared with WT cows (Additional file 1: Fig. S17a,b). The concentrations of the main proteins, such as BLG, bovine serum albumin (BSA), lactoferrin, and immunoglobulins had similar expression levels in milk from gene replacement cows and WT cows, while the expression level of bovine α-lactalbumin was decreased and casein was upregulated slightly (Additional file 1: Fig. S17c). The decreased expression of endogenous α-lactalbumin may be interfered by the expression of human α-lactalbumin, and the increased expression of casein may be caused by the destruction of single allele, leading to the compensatory effect. Above all, these results suggested that the SEGCPN method is a rapid and easy method for the generation of genetically humanized bovine.

**Table 1:** Expression of HLA in gene-replacement cows

α-LA	Gene-replacement cows (mean ± SD, g/L)	
	Cow 1	Cow 2
Human α-LA	1.60 ± 0.08	1.51 ± 0.03

## Discussion

In this study, we detailed a novel SEGCPN method that allowed the generation of fetuses and bovines with various types of precise gene editing, including point mutations, targeted deletions, gene tagging, and large gene fragment replacements, which have not been previously reported in large farm animals.

Gene targeting based on homologous recombination in which antibiotic resistance genes are used to select transgenic cells as donor cells for successful SCNT is the most effective method for producing transgenic farm animals. Selective marker genes (SMGs) are required for cell screening but need to be completely removed from bovine preimplantation embryos to achieve precision gene editing. Therefore, we must employ a promoter that is ubiquitously and specifically active in early embryos. In contrast to other embryonic factors, such as *Nanog* and *Sox2*, which are expressed in a mosaic pattern in preimplantation embryos, m*Oct4* is expressed in all embryonic cells until the late blastocyst stage and gradually disappears from the trophectoderm (TE) thereafter [30, 31]. Previous studies have indicated that the 18-kb m*Oct4* promoter can drive *EGFP* expression in both the inner cell mass (ICM) and TE of pig and cattle preimplantation embryos [32, 33]. However, the 18-kb *Oct4* promoter is too large for a number of molecular vectors. In this study, we chose to use a truncated m*Oct4* promoter including the core CR1-CR4 elements [34] as the embryonic promoter for the first time and constructed the ESSEE based on it. We found that the ESSEE functioned properly, remained active for cell screening, and performed self-excision in embryos. Importantly, we did not find mosaicism in various tissues of the fetuses or bovines with precise gene editing. In the future, smaller embryo-specifical promoters can be screened to reduce the size of the vector, which will be conducive to various operations in the later period.

One advantage of our technology is that it is versatile and generic; that is, the targeting vector including the ESSEE combined with programmable nucleases can be used for a variety of precise gene editing, such as point mutation, targeted deletion, small insertions, or gene replacements. Although gene knockout and knock-in pigs and cows can be produced using programmable nucleases, including zinc-finger nucleases (ZFNs) [35–37], TALENs [6], and Cas9 nuclease [7, 38, 39], these technologies present certain disadvantages, such as the generation of mosaicism of modification [39], the presence of selectable markers [6, 7, 36], and the natural point mutations and targeted deletions cannot usually be simulated. Although BE3 or BE4 technology has been successfully used to generate point mutations in pigs [15–17], goats [18], and sheep [19], other subtle mutations cannot be achieved with this technology, and non-C-to-T conversions are always induced [40, 41]. Moreover, the BE4 system causes many off-target effects in both RNA [42, 43] and DNA [44, 45]. The PE system, which allows the generation of targeted small insertions, deletions, and all types of point mutations, was shown to function efficiently in mammalian cells, plants, and mice [5, 20, 21]. However, its use has not been reported in large farm animals, and it cannot complete the insertion and replacement of long fragments. Our SEGCPN can simulate natural mutations, achieving point mutations and the full spectrum of nucleotide mutations at any position. Additionally, it can perform gene tagging and large fragment gene replacement in bovine with more precision than was achieved in a recent study, in which pigs with gene replacement were obtained by using Cas9 and dsDonor without the positive–negative marker, but with the disadvantage of inefficient and random insertion [22].

The other advantage of our technology is its high efficiency. Screening a moderate number of clones is sufficient to isolate a cell line containing the expected editing. Meanwhile, when the positive cells selected with this technology were employed as donors for in vitro embryo reconstruction, the blastocyst rate was normal, and embryos or bovine were produced normally. For point mutation and the 11-bp targeted deletion, the gene editing efficiencies were 64.6% and 81.8%, respectively. When these positive cells were used as donors for in vitro embryo reconstruction, the blastocyst rates ranged from 17.1 to 21.4%, with an average successful blastocyst rate of 19.3%, corresponding to normal efficiency. For gene tagging, the gene-targeting efficiency was 68.3%. When these positive cells were used as donors for in vitro embryo reconstruction, the blastocyst rate was 23.8%, which was a normal efficiency. Finally, for large gene fragments, the gene replacement efficiency was 57.1%. When these positive cells were used as donors for in vitro embryo reconstruction, the blastocyst rate was 21.5%, which was a normal efficiency.

Although the results of applying the SEGCPN method were surprising and counterintuitive, this approach still has some disadvantages. For example, this technology is currently only applicable to somatic cell cloning methods and is not suitable for cytoplasmic injection, which was a good strategy for producing gene-edited animals, including rodents [46, 47], zebrafish [48], rabbits [49], pigs [50], and bovine [51]. Besides, SCNT itself is limited by technical challenges, such as the finite proliferative capacity of the fetal fibroblasts used as donors for SCNT, the abnormal animals sometimes generated by SCNT, and the low efficiency of producing offspring by SCNT [52, 53]. However, SCNT has incomparable advantages over the cytoplasmic injection method, such as the risk of mosaicism is eliminated by using SCNT, and SCNT seems more suitable for the production of the KI gene-edited livestock. As reported in the present study, nearly 70% of the published KI farm animals were conducted using SCNT. Additionally, about half of the published KO farm animals were generated using SCNT [54]. Therefore, the development of SCNT and the improvement of SCNT efficiency [55] may further accelerate the application of our technology.

Additionally, our technology uses TALENs or Cas9, which can cause potential off-target effects [56, 57]. Recently, many strategies have been developed to reduce off-target mutagenesis and improve targeting precision [58], which may improve the SEGCPN method in the future. Much additional research is needed to further improve SEGCPN for broad application among different organisms and to increase the homozygous precise gene editing efficiency. Although a large number of clones can be screened to obtain homozygous gene knock-in-positive cells, the efficiency is relatively low. With the development of new methods that allow more efficient HDR-mediated targeting by inhibiting NHEJ [59, 60] or modifying the donor [61], we are likely to further increase the efficiency of our SEGCPN system.

In summary, we established an ESSEE in bovine for the first time; moreover, we established a versatile SEGCPN system based on this element. The system improved the traditional gene targeting and overcame major technological hurdles, such as point mutation, targeted deletion, gene tagging, and large gene fragment replacement. These findings give rise to additional possibilities for genome engineering in livestock for agricultural and biomedical applications.

## Conclusions

Precise gene editing, including targeted deletion, gene tagging, and large gene fragment replacement, remains a challenge in large farm animals. Here, we for the first time established versatile self-excising gene-targeting technology in combination with programmable nucleases (SEGCPN) to efficiently generate various types of precise gene editing in bovine, including point mutations and 11-bp deletions at the *MSTN* locus, EGFP labeling at the *SRY* locus, and large gene fragment replacement in which the endogenous 18-kb α-casein gene was replaced with a 2.6-kb human α-lactalbumin gene. In addition, we showed that the semen from the EGFP-labeled bulls appeared green, and the humanized cows in which the endogenous 18-kb α-casein gene was replaced with a 2.6-kb human α-lactalbumin gene successfully express the human α-lactalbumin protein. These findings represent a major advance toward precise gene editing in livestock and open up unlimited possibilities of genome engineering in livestock for applications in agriculture and biomedicine.

## Methods

### Animals

The ovaries from slaughtered mature cows were collected from a local abattoir in Beijing, China. Mature control and SCNT-recipient cows were obtained from the Dairy Cow Center of Beijing. The experimental bovines were housed in stalls with free access to food and water. All the animal work in this study was approved by the Institutional Animal Care and Use Committee of the China Agricultural University under approval number SKLAB-2014–07-05. We performed all surgeries under sodium pentobarbital anesthesia, and all attempts were made to minimize animal suffering.

### Vector construction

To construct the pTmO2.5-EGFP vector, the 2.5-kb m*Oct4* promoter was amplified by PCR from C57BL/6 mouse tail genomic DNA by using Q5 (M0491S, NEB) and was cloned into the pEGFP-N1 vector (Clontech, 6085–1) by using AseI (R0526S, NEB) and KpnI (R3142S, NEB). To construct the pTmOC-S-Cre vector, the mOCT4-Cre-pA cassette was synthesized by Sangon Biotech, and the product was cloned into the pCAG-stop2-EGFP vector by using PacI (R0547S, NEB). To construct the universal gene-targeting vector including the ESSEE, the loxP-TmOCT4-Cre-pA-POlII-Neo-pA-loxp self-excision cassette was synthesized by Sangon Biotech and was cloned into the pPGKneoDTA vector using NotI (R3189S, NEB) and NheI (R3131S, NEB), resulting in pPGKneoDTA-ESSEE. To construct the pMSTN-ESSEE targeting vector, a 5-kb 5′ homologous arm sequence was amplified by PCR from the bovine genome and was cloned into the pPGKneoDTA-ESSEE vector using NotI (R3189S, NEB) and SacII (R0157S, NEB), resulting in 5HR-pPGKneoDTA-ESSEE. A 2-kb 3′ homologous arm sequence including the G.C-to-A.T point mutation was synthesized by Sangon Biotech and was cloned into the 5HR-pPGKneoDTA-ESSEE vector using SalI (R3138S, NEB) and NheI (R3131S, NEB), resulting in pMSTN-ESSEE (point mutation). Similarly, A 2-kb 3′ homologous arm sequence including an 11-bp deletion was synthesized by Sangon Biotech and was cloned into the 5HR-pPGKneoDTA-ESSEE vector using SalI (R3138S, NEB) and NheI (R3131S, NEB), resulting in pMSTN-ESSEE (11-bp deletion). To construct the pSRY-ESSEE-EGFP vector, an 843-bp 5′ homologous arm and a 910-bp 3′ homologous arm were amplified by PCR from the genome of male BFFs and cloned into the pPGKneoDTA-ESSEE vector. The T2A-EGFP element was synthesized by Sangon Biotech. To construct the pHLA-ESSEE-RE vector, a 2-kb 5′ homologous arm and a 2-kb 3′ homologous arm were amplified by PCR from the genome of female BFFs and cloned into the pPGKneoDTA-ESSEE vector. The 2.6-kb replacement sequence consisted of *LALBA* cDNA and was synthesized by Sangon Biotech. Golden Gate assembly was used to construct the pSRY-ESSEE-EGFP and pHLA-ESSEE-RE vectors. To construct the CRISPR/Cas9 expression vectors, candidate sgRNAs were designed using the online software programs, and each 20-bp target sequence was sub-cloned into a pX330 vector (Addgene 42230). The CRISPR/Cas9 target sequences (20-bp target and 3-bp PAM sequence) used in this study are listed in Additional file 2: Table S13. All the vectors were confirmed through restriction digestion and Sanger sequencing. TALEN expression constructs were assembled by Viewsolid Biotech Company (Beijing, China). The characteristics of each TALEN pair are provided in Additional file 2: Table S14.

### Cell culture

BFFs were isolated from Holstein cattle fetuses and Angus cattle fetuses by disaggregating the entire body with the exception of the head and viscera and were cultured in Dulbecco’s modified Eagle’s medium (DMEM; Gibco, Grand Island, NY, USA) supplemented with 10% fetal bovine serum (FBS; Gibco, Grand Island, NY, USA) at 37.5 °C in an atmosphere of 5% CO_2_ and humidified air.

### Transfection

Primary fetal fibroblasts were thawed and cultured for 2 days to 80% subconfluence before transfection. The pTmO2.5-EGFP, pTmOC-S-Cre, pMSTN-ESSEE-KO, pSRY-ESSEE-EGFP, and pHLA-ESSEE-RE vectors were linearized with BstBI, AhdI, AscI AhdI, and AhdI, respectively, and then purified for transfection. To generate stable TmO2.5-EGFP and TmO2.5-Cre knock-in cells, BFFs (1 × 10^6^) were nucleofected with 3 µg of the pTmO2.5-EGFP vector or 3 µg of the pTmOC-S-Cre vector. To generate *MSTN*-edited cells, 4 µg of TALEN-M1 pair and 4 µg of the linearized donor pMSTN-ESSEE-KO were co-nucleofected into BFFs (1 × 10^6^). To generate pSRY-ESSEE-EGFP-targeted cells, 4 µg of Cas9-1 and 4 µg of the linearized donor pSRY-ESSEE-EGFP were co-nucleofected into BFFs (1 × 10^6^). To generate pHLA-ESSEE-RE-targeted cells, 4 µg of TALEN-C3 pair, 4 µg of TALEN-C6 pair, and 4 µg of the linearized donor pHLA-ESSEE-RE were co-nucleofected into BFFs (1 × 10^6^). The nucleofection procedure was according to the Amaxa Nucleofector System manufacturer’s guidelines (Lonza Group AG, Basel, Switzerland). T-016 program was selected. After 48 h, the transfected cells were transferred to 10-cm plates with 10% FBS containing G418 (1 mg/ml) at a density of approximately 1 × 10^5^ cells/plate. Individual cell clones were isolated 7–14 days after G418 selection, expanded, sequenced, and cryopreserved after a total of 12–14 days in culture.

### T7EI assay

The editing activity of each TALEN or Cas9 vector was assayed using T7EI (NEB) as described previously [62]. Briefly, genomic DNA from TALEN-treated or Cas9-treated cells was extracted using a DNeasy Blood and Tissue kit (Qiagen). PCR amplicons including nuclease target sites were generated using the following primer pairs: MSTN-F/MSTN-R for the *MSTN* locus, SRY-F/SRY-R for the *SRY* locus, CSN1-E1-F/CSN1-E1-R for the *CSN1* exon 1, and CSN1-E18-F/CSN1-E18-R for the *CSN1* exon 18. All the primers are listed in Additional file 2: Table S15. The PCR products were then denatured, rehybridized, digested with T7EI, and analyzed by agarose gel electrophoresis. The mutation frequencies (% indels) were calculated by measuring the band intensities. The bands were quantified based on the relative band intensities using the ImageJ software.

### Identification of positive cell clones by PCR

To identify positive cell clones, genomic DNA was extracted from single cell clones using a DNeasy Blood and Tissue kit (Qiagen). To confirm the successful *MSTN*-edited clones, P1/P2/P3 were used and were able to distinguish the targeted *MSTN* allele and the WT allele, as the P1/P3 primers amplified a 2.1-kb WT product, and the P2/P3 primers amplified a 2.5-kb targeted product. To confirm the successful production of pSRY-ESSEE-EGFP-targeted cells, two pairs of primers located between the donor and outside of the 5′ or 3′ homologous arm were used: the P6/P7 primer pair was used for the 5′ arm and produced a 2.1-kb amplicon, and the P8/P9 primer pair was used for the 3′ arm and produced a 1.9-kb amplicon. To confirm the successful generation of pHLA-ESSEE-RE-targeted cells, two pairs of primers located between the donor and outside of the 5′ or 3′ homologous arm were used: the P14/P15 primer pair was used for the 5′ arm and produced a 2.7-kb amplicon, and the P16/P17 primer pair was used for the 3′ arm and produced a 2.7-kb amplicon. The PCR procedure was performed using LA-Taq (Takara), with initial DNA denaturation at 94 °C for 5 min, followed by 30 cycles of 94 °C for 30 s, 60 °C for 30 s, and 72 °C for 2–8 min and a final 10-min extension. The PCR products were sequenced by TA cloning. All the primers are listed in Additional file 2: Table S15.

### Production of cloned embryos and bovine

The SCNT procedure was performed as described previously [63]. Briefly, transgenic cells were transferred into enucleated oocytes for the production of reconstructed embryos in vitro and were then fused using the ECM® 2001 Electro Cell Manipulation System (BTX, San Diego, CA, USA). The reconstructed embryos were activated with 10 mg/ml cycloheximide and 2.5 mg/ml cytochalasin-D in CR1aa medium. Day 7 blastocysts were collected for future transplantation. One to two reconstructed blastocysts were transferred into each recipient. Pregnancy was detected by ultrasonography at 60 days and 180 days posttransfer.

### Identification of fetuses or bovine

For the *MSTN* point mutation blastocysts or fetuses, one pair of primer P4/P5 was used, the heterozygous *MSTN*-edited blastocysts or fetuses produced a 1044-bp targeted band and a 960-bp WT band, the homozygous *MSTN*-edited blastocysts or fetuses only produced the 1044-bp targeted band. For the 11-bp *MSTN* deletion blastocysts or fetuses, the primer pair P4/P5 was used, the heterozygous *MSTN*-edited blastocysts or fetuses produced a 1033-bp targeted band and a 960-bp WT band, and the homozygous *MSTN*-edited blastocysts or fetuses only produced the 1033-bp targeted band. The PCR products were sequenced to confirm the point mutation or 11-bp deletion.

For the EGFP-labeled bulls, three pairs of primers, P6/P7, P10/P11, and P6/P11, were used, and these primer sets produced 2.2-kb, 1.9-kb, and 3.8-kb amplicons, respectively. For the site-specific gene replacement cows, three pairs of primers, P14/P15, P17/P18, and P14/P17, were used, and these primer sets produced 2.7-kb, 2.6-kb, and 6.8-kb amplicons, respectively. The PCR procedure was performed using LA-Taq (Takara), with initial DNA denaturation at 94 °C for 5 min, followed by 30 cycles of 94 °C for 30 s, 60 °C for 30 s, and 72 °C for 2–8 min and a final 10-min extension. All the primers are listed in Additional file 2: Table S15.

### Southern blotting

Genomic DNA was extracted from animal ear tissue using phenol/chloroform. At least 10 μg of genomic DNA from transgenic and WT bovine was digested with a restriction enzyme overnight. The digested genomic DNA was subjected to gel electrophoresis (0.7% agarose gel) and then transferred via the capillary method onto positively charged nylon membranes (Roche). The Southern blotting probe was amplified using the PCR DIG Probe Synthesis Kit (Roche), with the EGFP-DIG-F/EGFP-DIG-R primers for the EGFP-labeled bulls and the HLA-DIG-F/HLA-DIG-R primers for the gene replacement cows. The primers are listed in Additional file 2: Table S15. Prehybridization and hybridization were performed at 45 °C, and washing steps were performed at 68 °C. For the EGFP-labeled bulls, the positive bands were expected to be 8.4 kb, 4.0 kb, and 3.3 kb when digested by the BglI, PstI, and BsrGI restriction enzymes, respectively. For the site-specific gene replacement cows, the positive bands were expected to be 8.2 kb and 4.4 kb when digested by SpeI and BamHI restriction enzymes, respectively.

### RT-PCR

Total mRNA was extracted from various adult tissues using TRIzol Reagent (Life Technologies) prior to performing RT-PCR analyses. A total of 300 ng of total RNA was reverse transcribed. The primers P12/P13 were used to amplify the SRY-EGFP, and the primers GAPDH-F/GAPDH-R were used to amplify the bovine glyceraldehyde-3-phosphate dehydrogenase (GAPDH) gene as a control. The expected PCR products of SRY-EGFP and GAPDH were 888 bp and 488 bp, respectively. The primers are listed in Additional file 2: Table S15.

### Identification of recombinant HLA

The lactation of transgenic and non-transgenic cows (6 to 8 mo of age) was induced with lactating agents (National Caotan Pharmacy Company, Xi’an, China). For Native-PAGE, milk protein samples were separated on 15% Tris–glycine polyacrylamide gels under non-denaturing conditions, and the protein contents were quantified by dying the gels with Coomassie brilliant blue. For Western blotting analysis, diluted milk samples were separated on 15% Tris–glycine polyacrylamide gels under non-denaturing conditions and were then transferred to polyvinyl difluoride membranes (Invitrogen Corporation, Carlsbad, CA, USA), which were incubated with a polyclonal anti-HLA antibody (dilution, 1:500; Sigma) and a horseradish peroxidase (HRP)-conjugated secondary anti-rabbit IgG antibody (dilution, 1:10,000; Sino-American Co.). The HLA expression levels were measured using a Human α-lactalbumin Enzyme-linked Immunosorbent Assay (ELISA) Kit (Abcam, Cambridge, MA, USA).

### Analysis of milk components

Milk from gene-replacement and WT type of the same age cows were collected at 3 different time points. The gross composition of whole milk samples from the two types of cows was determined using MilkoScan 4000 (Foss Electric, Hillerod, Denmark) for analysis of the percentage of total fat, total protein, total lactose, and total solids. The main proteins (BLG, casein, BSA, lactoferrin, and immunoglobulin) in the milk were detected using commercial ELISA kits (eBioscience, Inc., San Diego, CA).

### Hematoxylin and eosin (H&E) staining

The tissues were fixed with 4% paraformaldehyde for 48 h, embedded in paraffin wax, sectioned onto slides, stained with H&E, and then analyzed by microscopy (Nikon TS100). For each sample, five view fields (areas) were randomly selected and then analyzed using the Image Pro Plus 6.0 software. The cross-sectional areas of 100–200 myofibers were measured, and the average area of each myofiber and diameter of myofibers were determined.

### Immunofluorescent analysis

The tissues fixed with 10% formalin for 30 min at room temperature and frozen in optimal cutting temperature compound (OCT, Fisher Healthcare, Waltham, MA, USA). Sections 3–5 µm thick were mounted on glass slides. The sections were permeabilized and blocked for 1 h at room temperature (0.25% Triton X-100, 4% goat serum in PBS). Then, incubation with primary antibodies (GFP, 1:100, Abcam, Cambridge, MA, USA) was done in blocking solution (4% goat serum in PBS) at 4 °C overnight. Secondary antibodies were applied in blocking solution for 1 h at room temperature. Samples were mounted with VectaShield mounting medium with DAPI (Vector Laboratories, Burlingame, CA, USA).

### Prediction of off-target sites

For each TALEN pair off-target prediction, the genome sequence (Latin name: Bos Taurus, reference genome version: Ruminant Reference Genome, 4.6.1/bos Tau7) was divided into a small units of 10 kb. The left and right sequences of TALEN pairs were input into the Cutadapt linkedAdpter program (https://cutadapt.readthedocs.io/en/stable/guide.html#linked-adapters) with full-length matching, no indels allowed, and a maximum base mismatch of 5. LinkedAdpter cyclically searched within each segmented small unit; that was, after a successful match, it searched again from the position after the match until another match could not be found. In the matching process, only results with spacing lengths greater than 10 and less than 30 were retained. For the sgRNA off-target prediction, an online tool Cas-OFFinder (https://www.rgenome.net/cas-offinder) was used. All primers for off-target assay are listed in Additional file 2: Table 15.

### Supplementary Information

**Additional file 1: Fig. S1. **Establishment of the embryo-specific self-excising element.** Fig. S2. **Design of TALENs for the induction of DNA double-strand breaks (DSBs) in the endogenous MSTN gene. **Fig. S3. **DNA sequencing of the MSTN G.C-to-A.T point mutation.** Fig. S4. **Off-target analysis of MSTN TALENs-M1 in fetus.** Fig. S5. **DNA sequencing of the 11-bp MSTN deletion. **Fig. S6. **H&E staining of the muscle from WT and MSTN homozygous 11-bp deletion fetus. **Fig. S7. **Design of Cas9 for the induction of DSBs in the endogenous SRY gene.** Fig. S8. **Identification of pSRY-ESSEE-EGFP knock-in cell clones generated by Cas9-mediated gene homologous recombination at the SRY locus. **Fig. S9. **Off-target analysis of SRY sgRNA-1 in the pSRY-EGFP bulls.** Fig. S10. **Immunohistochemical analysis of testis.** Fig. S11. **Design of TALENs for the induction of DSBs in the endogenous CSN1 exon 1.** Fig. S12. **Design of TALENs for the induction of DSBs in the endogenous CSN1 exon 18. **Fig. S13. **Identification of pHLA-ESSEE-RE knock-in cell clones by TALEN-mediated gene replacement.** Fig. S14. **Analysis of the WT allele for the site-specific gene replacement cows.** Fig. S15. **Off-target analysis of CSN1 TALENs-C3 in the gene-replacement cows.** Fig. S16. **Off-target analysis of CSN1 TALENs-C6 in the gene-replacement cows.** Fig. S17. **Analysis of milk composition.

**Additional file 2: Table S1.** Summary of the PCR results of G418-resistant pMSTN-ESSEE (point mutation)-targeted clones.** Table S2. **Summary of the NT results of pMSTN-ESSEE (point mutation)-targeted BFFs. **Table S3. **List of the top 4 potential off-target effects of MSTN TALENs-M1.** Table S4. **Summary of the PCR results of G418-resistant pMSTN-ESSEE (11-bp deletion)-targeted clones.** Table S5. **Summary of the NT results of pMSTN-ESSEE (11-bp deletion)-targeted BFFs.** Table S6. **Summary of the PCR results of G418-resistant EGFP-targeted clones.** Table S7. **Summary of the nuclear transfer results of EGFP-targeted BFFs.** Table S8. **List of the top 4 potential off-target effects of SRY Cas9-sgRNA1.** Table S9.** Summary of the PCR results of G418-resistant gene-replacement clones.** Table S10. **Summary of the nuclear transfer results of gene-replacement BFFs. **Table S11. **List of the top 4 potential off-target effects of CSN1 TALENs-C3. **Table S12. **List of the top 4 potential off-target effects of CSN1 TALENs-C6.** Table S13. **Target sequences of the SRY sgRNAs used in this study.** Table S14. **TALEN recognition sequences and amino acid sequences of the repeat variable di-residues (RVDs) in the corresponding TALENs.** Table S15. **List of primers used in the present study.

**Additional file 3.** Supporting data. 

## Data Availability

All data generated or analyzed during this study are included in this published article and its supplementary information files. The authors state that all data necessary for confirming the conclusions presented in this article are represented fully within the article or can be provided by the authors upon request.

## Abbreviations

SEGCPN: Self-excising gene-targeting technology in combination with programmable nucleases
ESSEE: Embryo-specific self-excision element
BFFs: Bovine fetal fibroblasts
SCNT: Somatic cell nuclear transfer
WT: Wild type
TmO2.5: Truncated mOct4 promoter
DSBs: Double-strand DNA breaks
HDR: Homology-directed repair
PCR: Polymerase chain reaction
T7EI: T7 endonuclease I
sgRNA: Single-guide RNA
BE: Base editors
PE: Prime editing
