# Supplementary material for: Versatile generation of precise gene edits in bovines using SEGCPN
Source: BMC Biol. 2023 Oct 20;21:226. doi: 10.1186/s12915-023-01677-0 (PMC10589966; doi:10.1186/s12915-023-01677-0)
Supplement: Supplementary file 1 — Additional file 1: Fig. S1. Establishment of the embryo-specific self-excising element. Fig. S2. Design of TALENs for the induction of DNA double-strand breaks (DSBs) in the endogenous MSTN gene. Fig. S3. DNA sequencing of the MSTN G.C-to-A.T point mutation. Fig. S4. Off-target analysis of MSTN TALENs-M1 in fetus. Fig. S5. DNA sequencing of the 11-bp MSTN deletion. Fig. S6. H&E staining of the muscle from WT and MSTN homozygous 11-bp deletion fetus. Fig. S7. Design of Cas9 for the induction of DSBs in the endogenous SRY gene. Fig. S8. Identification of pSRY-ESSEE-EGFP knock-in cell clones generated by Cas9-mediated gene homologous recombination at the SRY locus. Fig. S9. Off-target analysis of SRY sgRNA-1 in the pSRY-EGFP bulls. Fig. S10. Immunohistochemical analysis of testis. Fig. S11. Design of TALENs for the induction of DSBs in the endogenous CSN1 exon 1. Fig. S12. Design of TALENs for the induction of DSBs in the endogenous CSN1 exon 18. Fig. S13. Identification of pHLA-ESSEE-RE knock-in cell clones by TALEN-mediated gene replacement. Fig. S14. Analysis of the WT allele for the site-specific gene replacement cows. Fig. S15. Off-target analysis of CSN1 TALENs-C3 in the gene-replacement cows. Fig. S16. Off-target analysis of CSN1 TALENs-C6 in the gene-replacement cows. Fig. S17. Analysis of milk composition. [file 12915_2023_1677_MOESM1_ESM.docx]

**Additional File1**

**Versatile generation of precise gene edits in bovines using SEGCPN**

Ming Wang^1,2,3^, Fangrong Ding^2^, Haiping Wang^2^, Ling Li^2^, Yunping Dai^2^^*^, ZhaoLin Sun^1,2,3*^,

Ning Li^2,3*^

^1^College of Animal Science and Technology, China Agricultural University, No.2 Yuanmingyuan Xilu, Beijing 100193, China.

^2^College of Biological Sciences, China Agricultural University, No.2 Yuanmingyuan Xilu, Beijing 100193, China.

^3^Beijing Capital Agribusiness Future Biotechnology Co., Ltd, No.75 Bingjiaokou Hutong, Beijing 100088, China.

^*^Correspondence and requests for materials should be addressed to Y.D. (email:[daiyunping@sina.com](mailto:daiyunping@sina.com)), Z. S. (email: [sunzhaolin@bjsnzz.com](mailto:sunzhaolin@bjsnzz.com)) or N.L. (lining@bjsngf.com.cn).

**FIGURES-Additional File**

**
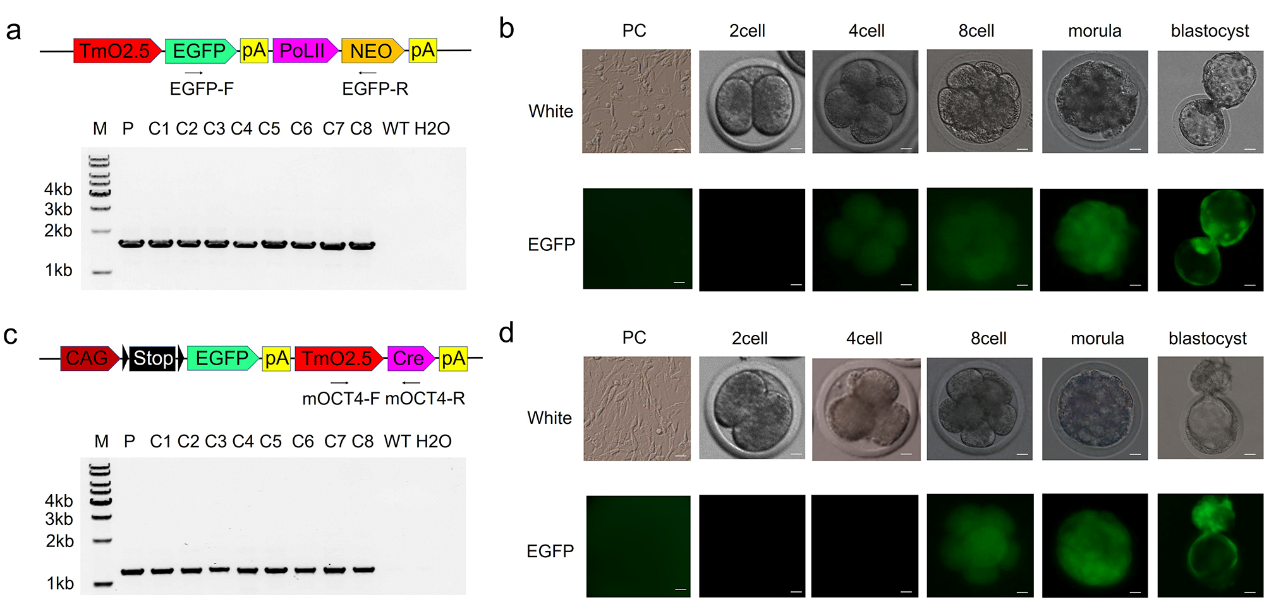
**

**Fig.S1 Establishment of the embryo-specific self-excising element**

**(a)** Diagram of the pTmO2.5-EGFP vector and PCR analysis of BFF clones stably transfected with pTmO2.5-EGFP. The pTmO2.5-EGFP vector contained the 2.5-kb truncated m*Oct4* (TmO2.5) sequence followed by the *EGFP* gene and a neomycin-resistance gene (NEO). EGFP-F and EGFP-R amplified a 1.8-kb product to confirm stable positive pTmO2.5-EGFP knock-in clones. M, 1-kb DNA ladder; C1–C8, BFF clones; P, pTmO2.5-EGFP plasmids; WT, wild- type cells; H_2_O, blank control. **(b)** EGFP expression in pTmO2.5-EGFP transgenic bovine embryos. PC: Positive cell clone as a control. **(c)** Diagram of the TmO2.5-Cre reporter vector (pTmOC-S-Cre) and PCR analysis of BFF clones stably transfected with pTmOC-S-Cre. The pTmOC-S-Cre reporter vector containing the *CAG* promoter followed by the STOP cassette flanked with two *loxP*s, the *EGFP* gene, TmO2.5, and the *Cre* gene. The mOCT4-F and mOCT4-R primers amplified a 1.2-kb product to confirm stable positive pTmOGN-S-Cre knock-in clones. M, 1-kb DNA ladder; C1–C8, BFF clones; P, pTmOC-S-Cre plasmids; WT, wild-type cells; H_2_O, blank control. **(d)** EGFP expression in pTmOC-S-Cre transgenic bovine embryos. PC: Positive cell clone as a control.

**
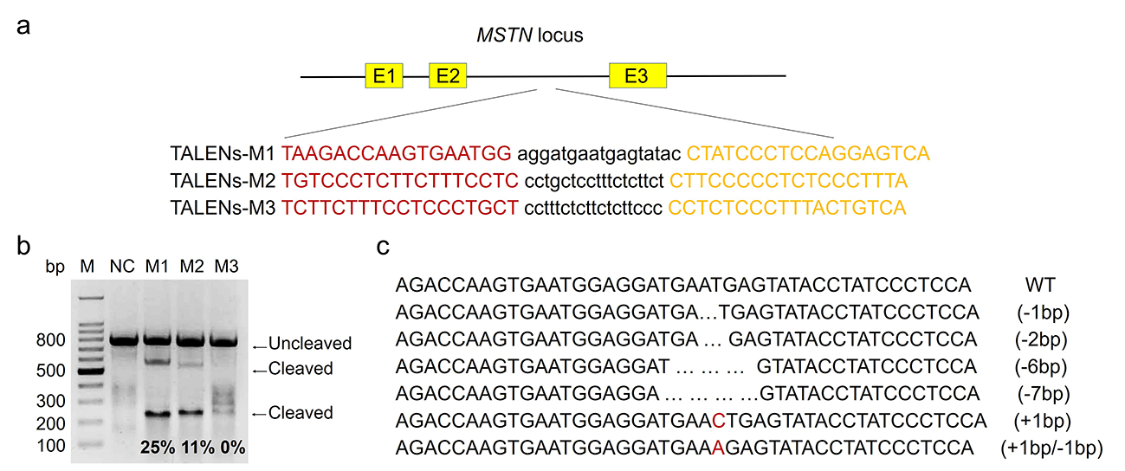
**

**Fig.S2 Design of TALENs for the induction of DNA double-strand breaks (DSBs) in the endogenous *MSTN* gene**

**(a)** Modification of the bovine *MSTN* gene by TALENs. The recognition sequences of the three TALEN pairs used in this study are highlighted: the left recognition sequence is highlighted in red, and the right recognition sequence is highlighted in yellow. E1–E3, exon 1–exon 3 of the *MSTN* gene. **(b)** Representative results of T7EI assays. The mutation frequencies (% indels) of different TALEN pairs were calculated by measuring the band intensities. The uncleaved and cleaved products are indicated. M, 100-bp DNA ladder; NC, wild-type control cells; M1, TALEN pair M1-transfected cells; M2, TALEN pair M2-transfected cells; M3, TALEN pair M3-transfected cells. **(c)** Representative sequencing results of the TA clones revealing different indel mutations mediated by TALENs-M1 at the target site.


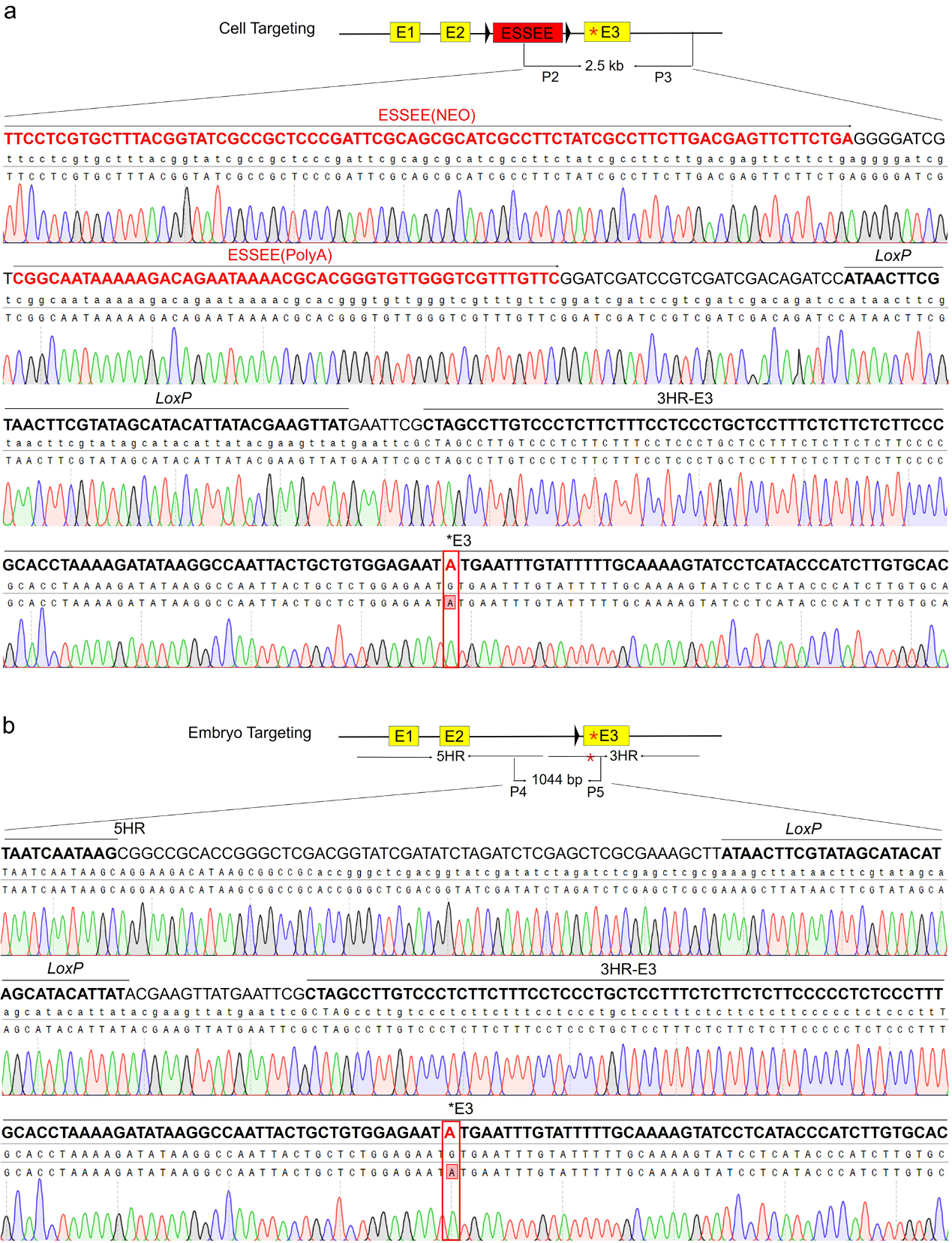


**Fig.S3 DNA sequencing of the *MSTN* G.C-to-A.T point mutation**

**(a)** DNA sequencing of *MSTN* G.C-to-A.T point mutation positive cell clones. **(b)** DNA sequencing of the *MSTN* G.C-to-A.T point mutation embryos.


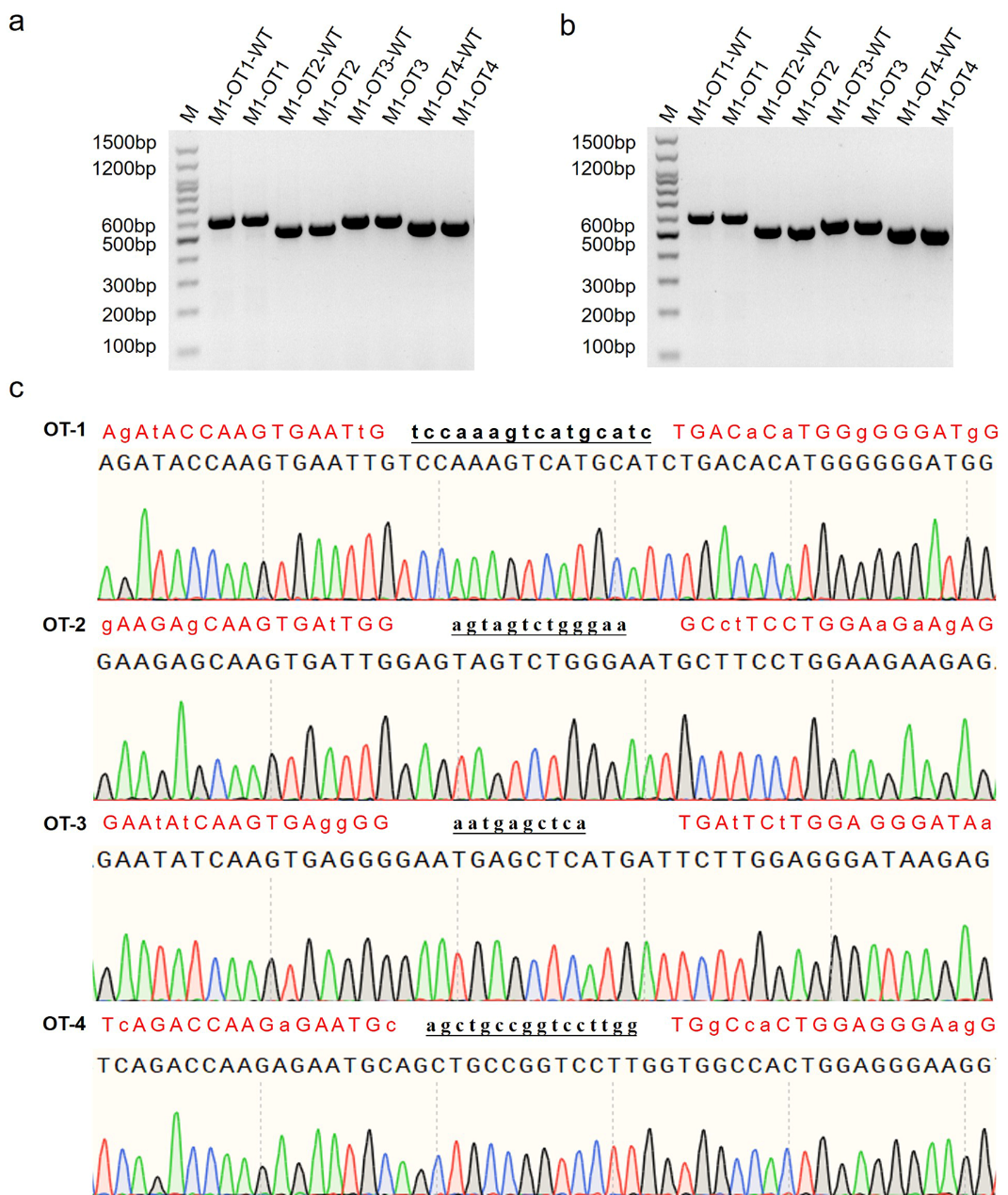


**Fig.S4 Off-target analysis of *MSTN* TALENs-M1 in fetus**

**(a)** T7EI cleavage analysis of the top 4 potential off-target sequences in the G.C-to-A.T point mutation heterozygous fetus. **(b)** T7EI cleavage analysis of the top 4 potential off-target sequences in the 11-bp deletion homozygous fetus. OT-1-OT-4, top 4 potential off-target sites; M, 100-bp DNA ladder; WT, genome from WT fetus; M1, genome from mutation fetus. **(c)** Representative chromatogram sequence analysis of the top 4 potential off-target sequences. The TALEN recognition sequences, including paired left and right recognition sequences (colored in red (uppercase indicates a match with the consensus; lowercase indicates a mismatch)) and the spacer sequences (underlined) between left and right hits, are shown.

**
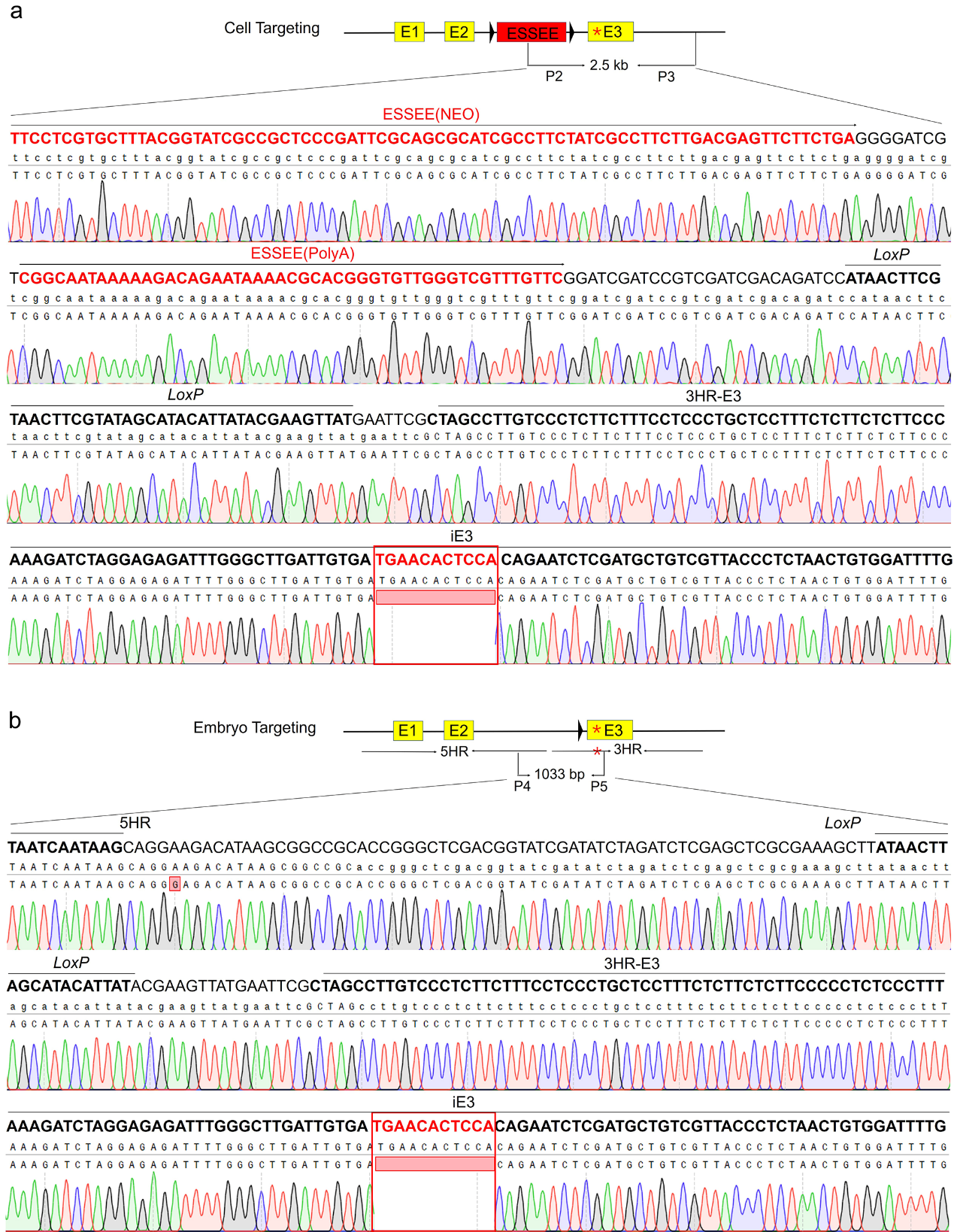
**

**Fig.S5 DNA sequencing of the 11-bp *MSTN* deletion**

**(a)** DNA sequencing of the 11-bp *MSTN* deletion positive cell clones. **(b)** DNA sequencing of the 11-bp *MSTN* deletion embryos.


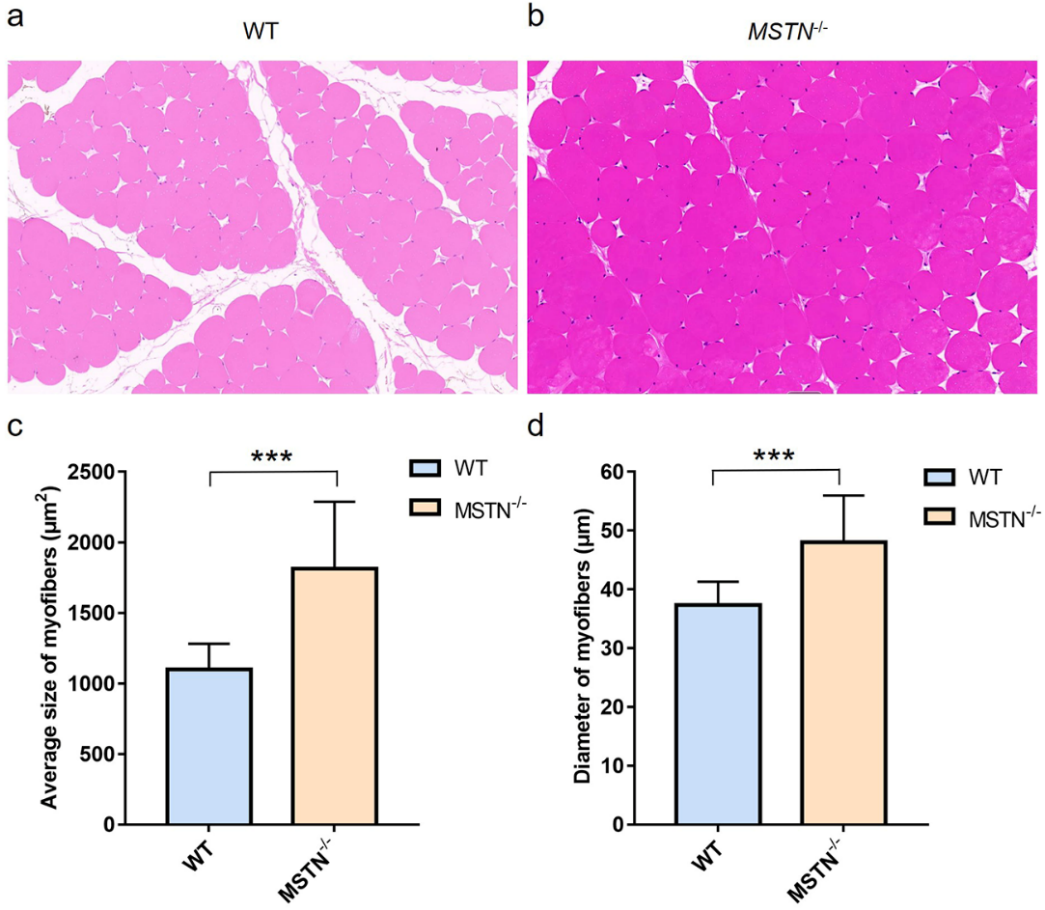


**Fig.S6 H****&E staining of the muscle from WT and *MSTN* homozygous 11-bp deletion fetus**

**(a)** H&E staining of the muscle from WT fetus. **(b)** H&E staining of the muscle from *MSTN* homozygous 11-bp deletion fetus. Scale bars = 100 µm. **(c)** The average size of muscle fibers from fetus with *MSTN* homozygous 11-bp deletion and WT. **(d)** The diameter of myofibers from fetus with *MSTN* homozygous 11-bp deletion and WT. light blue bar: WT; light orange bar: *MSTN^−/−^.* Data are expressed as mean±SEM. ***P < 0.001.


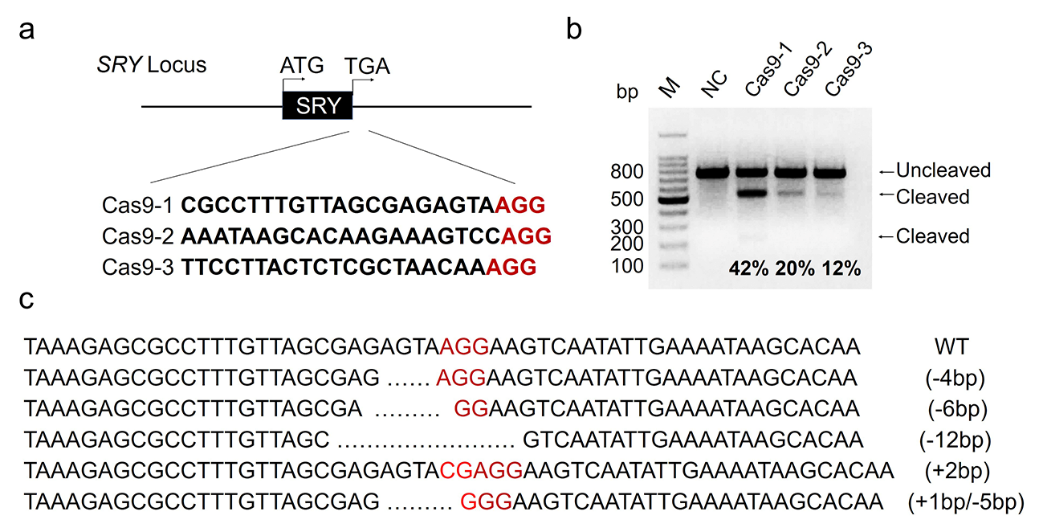


**Fig.S7 Design of Cas9 for the induction of DSBs in the endogenous *SRY* gene**

**(a)** Modification of the bovine *SRY* gene by CRISPR/Cas9. The CRISPR/Cas9 target sequences (20-bp target and 3-bp PAM sequence (colored in red)) are shown. **(b)** Representative results of T7EI assays. The mutation frequencies (% indels) of different sgRNAs were calculated by measuring the band intensities. The uncleaved and cleaved products are indicated. M, 100-bp DNA ladder; NC, wild-type control cells; Cas9-1, Cas9-1-transfected cells; Cas9-2, Cas9-2-transfected cells; Cas9-3, Cas9-3-transfected cells. **(c)** Representative sequencing results of the TA clones revealing different indel mutations mediated by Cas9-1 at the target site.

**
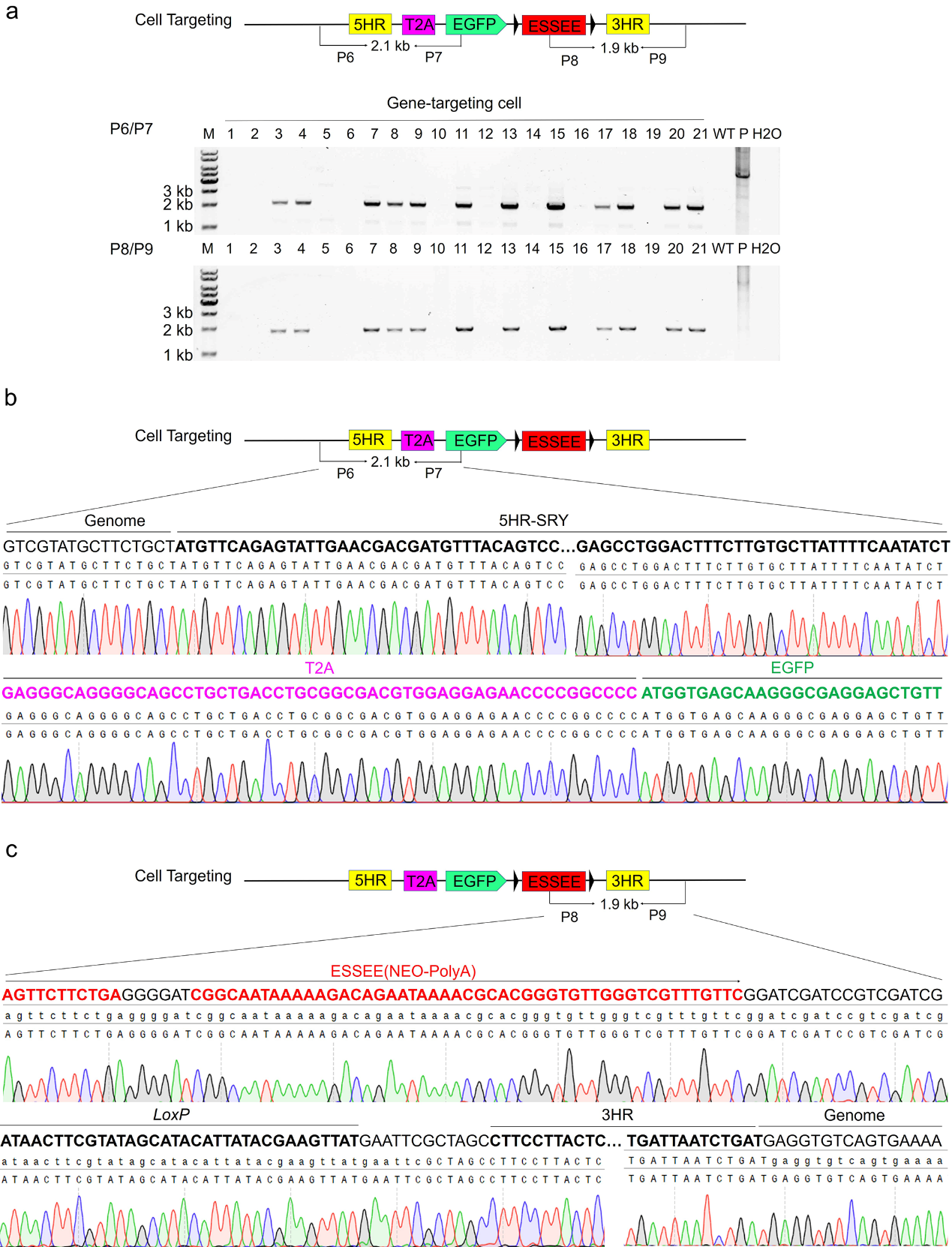
**

**Fig.S8 Identification of pSRY-ESSEE-EGFP knock-in cell clones generated by Cas9-mediated gene homologous recombination at the *SRY* locus**

**(a)** PCR analysis of pSRY-ESSEE-EGFP knock-in cells. The targeted introduction of pSRY-EGFP resulted in the amplification of the expected band of 2.1-kb from P6 and P7 and the expected band of 1.9-kb from P8 and P9. M, 1-kb DNA ladder; 1–21, G418-resistant cell clones; P, donor vector; WT, wild-type cells; H_2_O, negative control. **(b)** Sequencing confirmation of the 5′ junction after the targeted integration of pSRY-ESSEE-EGFP cassettes into the bovine *SRY* gene. **(c)** Sequencing confirmation of the 3′ junction after the targeted integration of pSRY-ESSEE-EGFP cassettes into the bovine *SRY* gene.


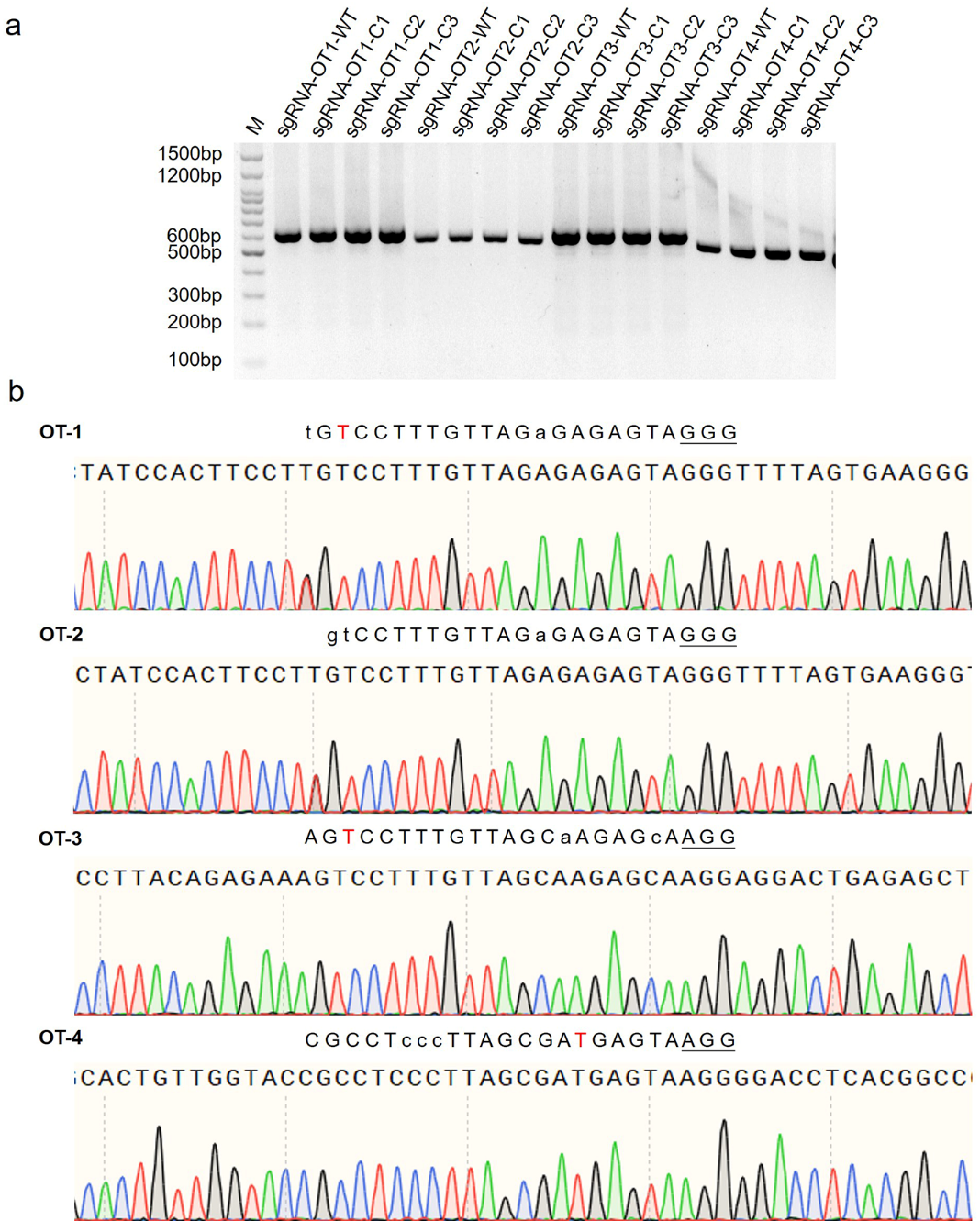


**Fig.S9 Off-target analysis of *SRY* sgRNA-1 in the pSRY-EGFP bulls**

**(a)** T7EI cleavage analysis of the top 4 potential off-target sequences in the pSRY-EGFP bulls. OT-1-OT-4, top 4 potential off-target sites; M, 100-bp DNA ladder; WT, genome from WT bull; C1-C3, genome from the pSRY-EGFP bulls. **(b)** Representative chromatogram sequence analysis of the top 4 potential off-target sequences. The 20-bp target sequences (uppercase indicates a match with the consensus; lowercase indicates a mismatch; colored in red indicates the bulge base) and 3-bp PAM sequences (underlined) are shown.


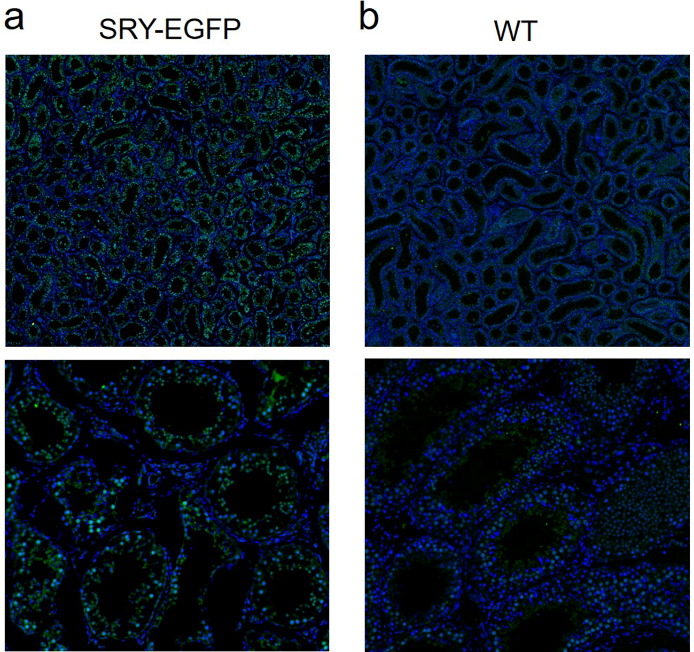


**Fig.S10 Immunohistochemical analysis of testis**

**(a)** Immunohistochemical analysis of SRY-EGFP bull’s testis (GFP). **(b)** Immunohistochemical analysis of SRY-EGFP bull’s testis (GFP). 1000 µm (up panel, 2x) and 100 µm (down panel, 10x).

**
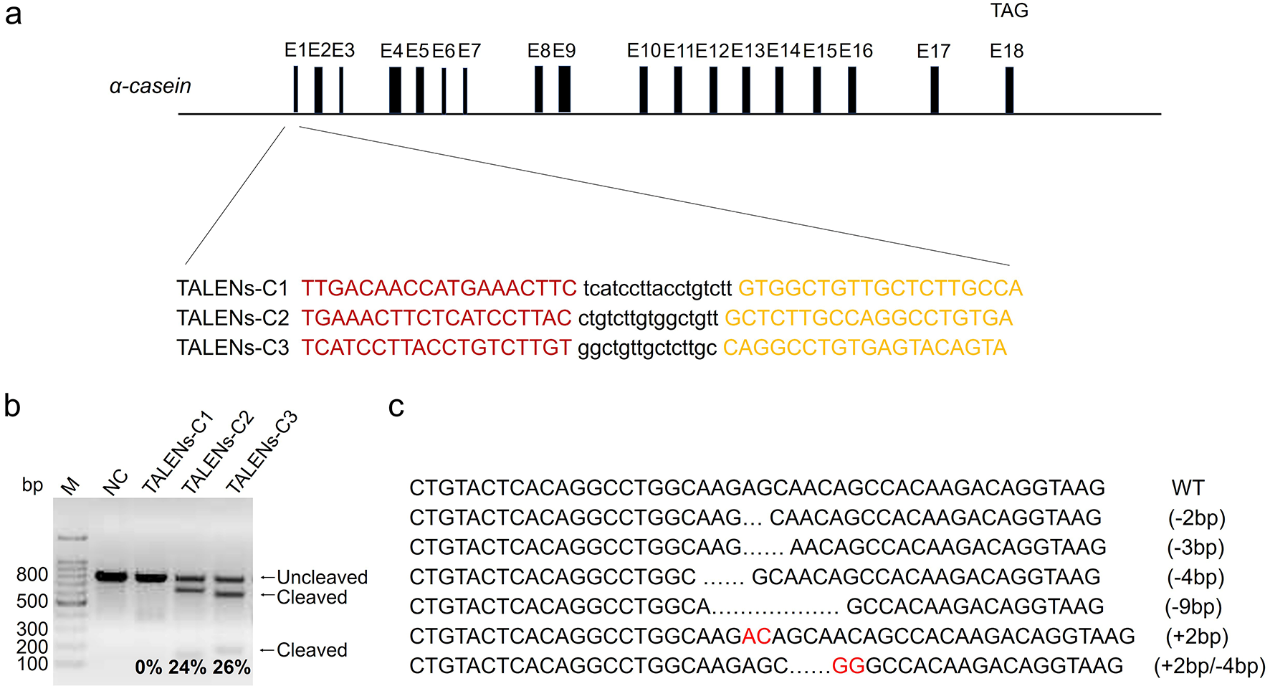
**

**Fig.S11 Design of TALENs for the induction of DSBs in the endogenous *CSN1* exon 1**

**(a)** Modification of the bovine *CSN1* exon 1 by TALENs. The recognition sequences of the three TALEN pairs used in this study are highlighted: the left recognition sequence is highlighted in red, and the right recognition sequence is highlighted in yellow. E1–E18, exon 1–exon 18 of the *CSN1* gene. **(b)** Representative results of T7EI assays. The mutation frequencies (% indels) of different TALEN pairs were calculated by measuring the band intensities. The uncleaved and cleaved products are indicated. M, 100-bp DNA ladder; NC, wild-type control cells; TALENs-C1, TALEN pair C1-transfected cells; TALENs-C2, TALEN pair C2-transfected cells; TALENs-C3, TALEN pair C3-transfected cells. **(c)** Representative sequencing results of the TA clones revealing different indel mutations mediated by TALENs-C3 at the target site.

**
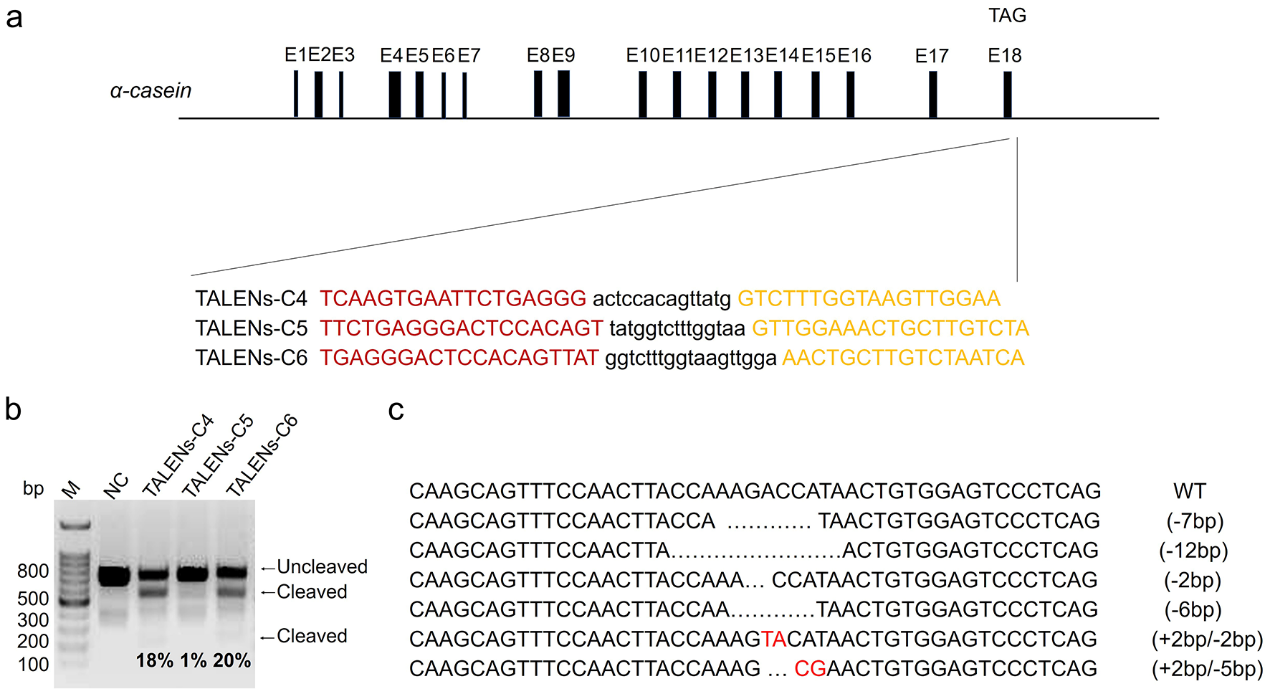
**

**Fig.S12 Design of TALENs for the induction of DSBs in the endogenous *CSN1* exon 18**

**(a)** Modification of the bovine *CSN1* exon 18 by TALENs. The recognition sequences of the three TALEN pairs used in this study are highlighted: the left recognition sequence is highlighted in red, and the right recognition sequence is highlighted in yellow. E1–E18, exon 1–exon 18 of the *CSN1* gene. **(b)** Representative results of T7EI assays. The mutation frequencies (% indels) of different TALEN pairs were calculated by measuring the band intensities. The uncleaved and cleaved products are indicated. M, 100-bp DNA ladder; NC, wild-type control cells; TALENs-C4, TALEN pair C4-transfected cells; TALENs-C5, TALEN pair C5-transfected cells; TALENs-C6, TALEN pair C6-transfected cells. **(c)** Representative sequencing results of the TA clones revealing different indel mutations mediated by TALENs-C6 at the target site.

**
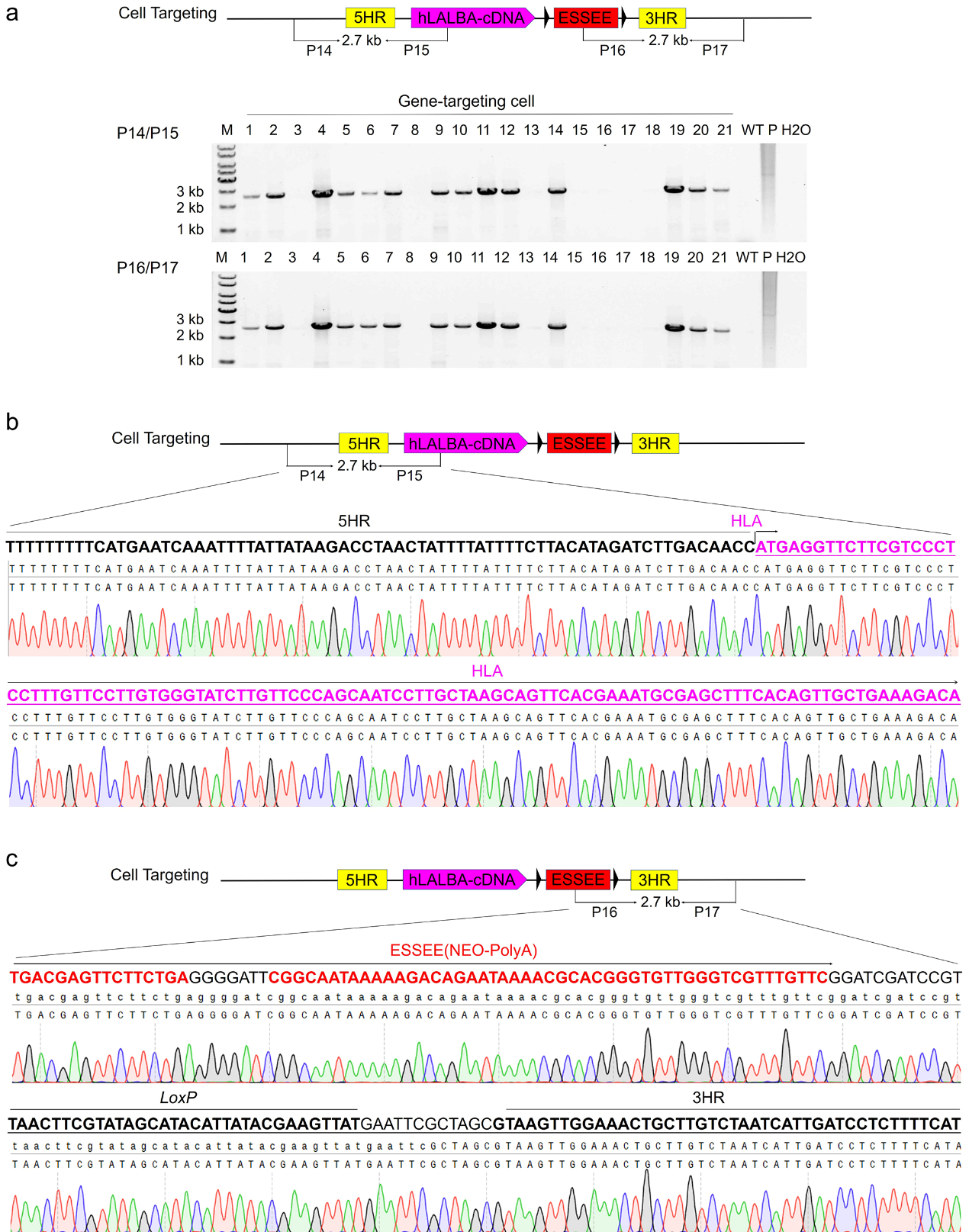
**

**Fig.S13 Identification of pHLA-ESSEE-RE knock-in cell clones by TALEN-mediated gene replacement**

**(a)** PCR analysis of the pHLA-ESSEE-RE knock-in cells. The targeted introduction of pHLA-ESSEE-RE resulted in the amplification of the expected band of 2.7-kb from P14 and P15 and the expected band of 2.7-kb from P16 and P17. M, 1-kb DNA ladder; 1–21, G418-resistant cell clones; P, donor vector; WT, wild type cells; H_2_O, negative control. **(b)** Sequencing confirmation of the 5′ junction after the targeted integration of pHLA-ESSEE-RE cassettes into the bovine *CSN1* gene. **(c)** Sequencing confirmation of the 3′ junction after the targeted integration of pHLA-ESSEE-RE cassettes into the bovine *CSN1* gene.

**
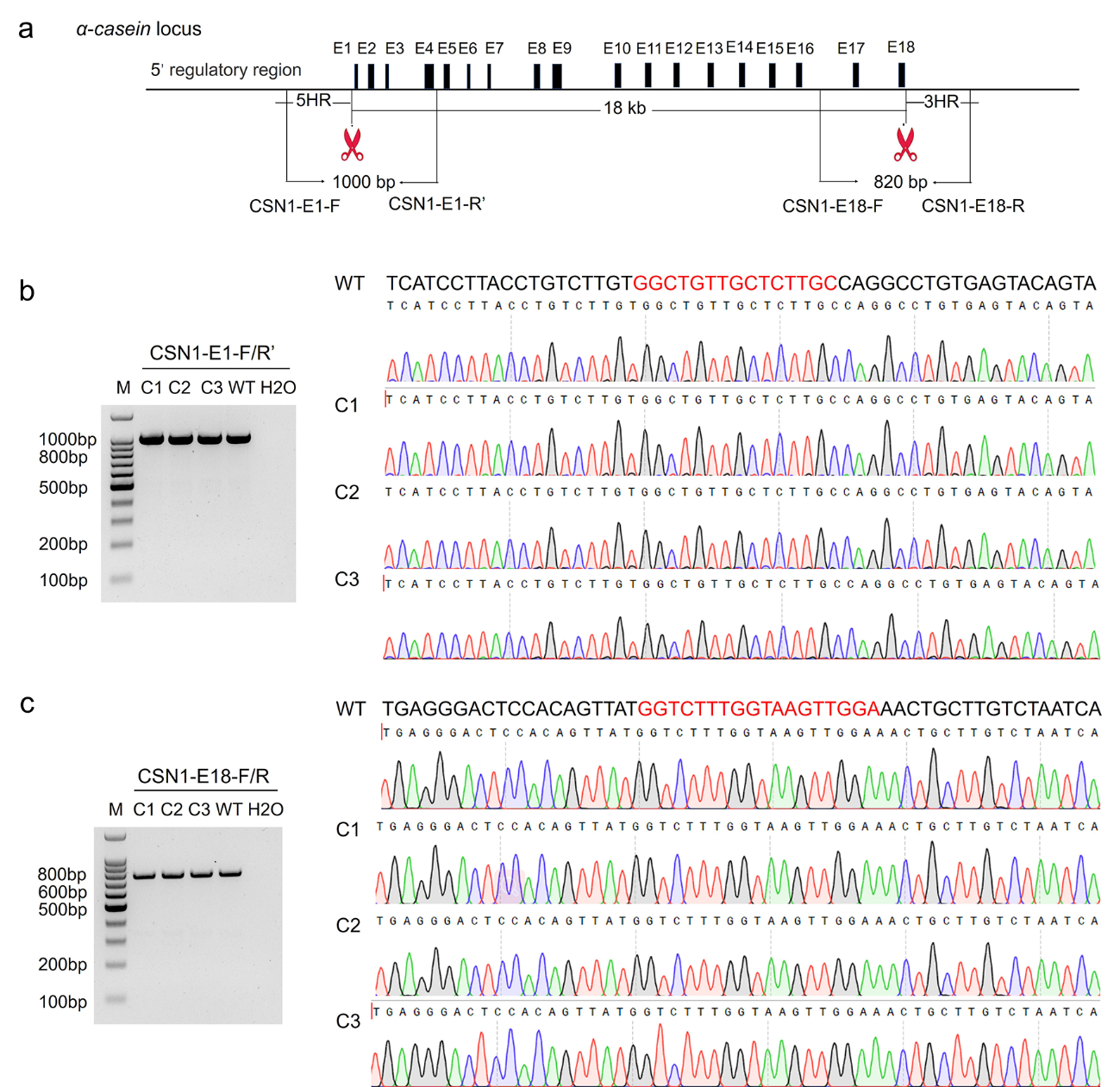
**

**Fig.S14 Analysis of the WT allele for the site-specific gene replacement cows**

**(a)** Schematic representation of the WT allele PCR analysis of the cows with site-specific gene replacement. Primers were designed upstream and downstream of the TALEN incision site for amplification and sequencing to confirm that the allele was the WT type. Primers CSN1-E1-F and CSN1-E1-R’ amplified a 1000-bp product, primers CSN1-E18-F and CSN1-E18-R amplified a 820-bp product. **(b)** PCR analysis and DNA sequencing with primers CSN1-E1-F/R’ for confirmation of the 5′ incision region. The left was the PCR analysis. M, 100-bp DNA ladder; C1–C3, gene replacement cows; WT, wild-type cow; H_2_O, negative control. The right was the DNA sequencing. The sequence colored in red was the incision site for TALEN. **(c)** PCR analysis and DNA sequencing with primers CSN1-E18-F/R for confirmation of the 3′ incision region. The left was the PCR analysis. M, 100-bp DNA ladder; C1–C3, gene replacement cows; WT, wild-type cow; H_2_O, negative control. The right was the DNA sequencing. The sequence colored in red was the incision site for TALEN.


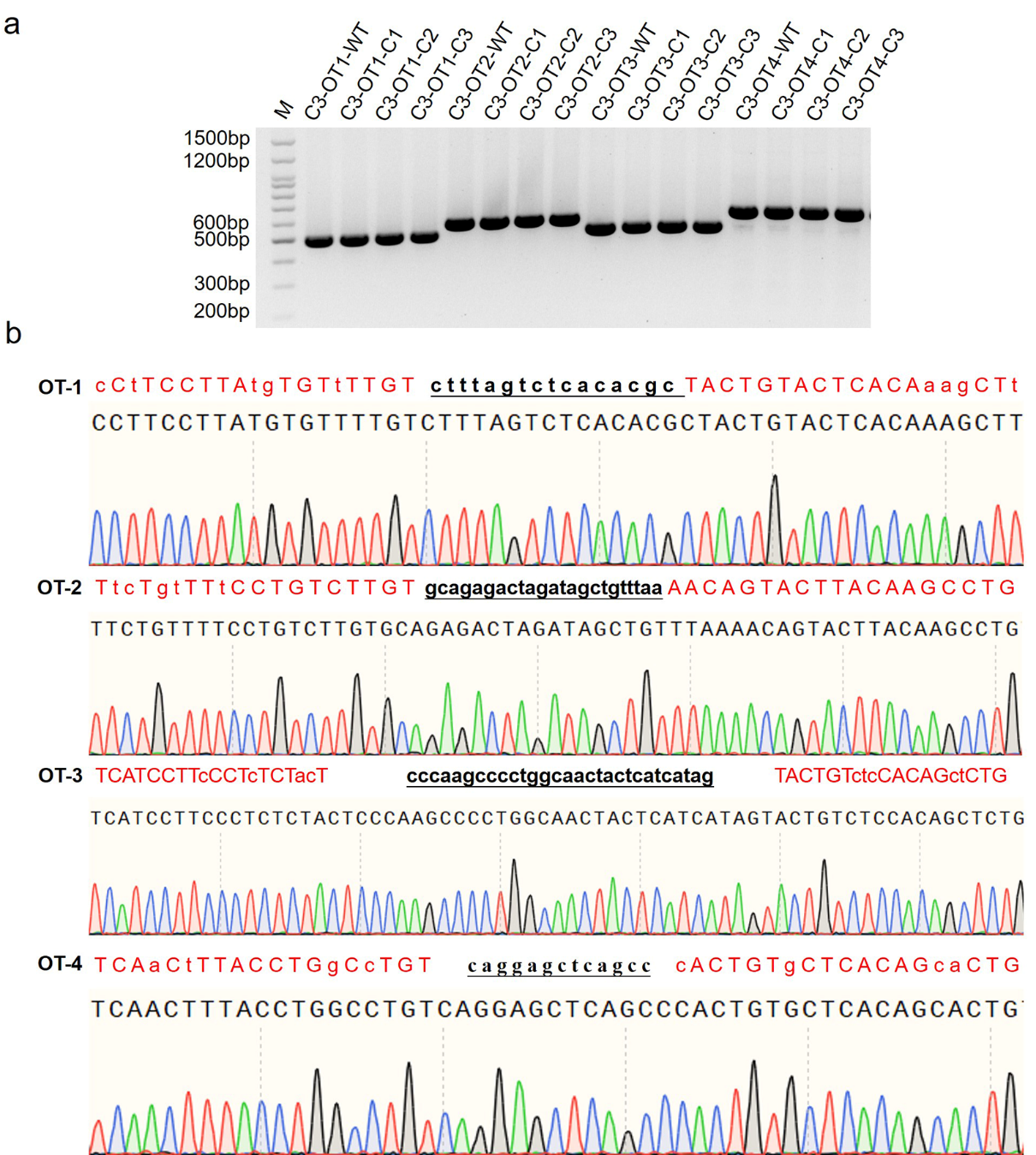


**Fig.S15 Off-target analysis of *CSN1* TALENs-C3 in the gene-replacement cows**

**(a)** T7EI cleavage analysis of the top 4 potential off-target sequences in the gene-replacement cows. OT-1-OT-4, top 4 potential off-target sites; M, 100-bp DNA ladder; WT, genome from WT cow; C1-C3, genome from the gene-replacement cows. **(b)** Representative chromatogram sequence analysis of the top 4 potential off-target sequences. The 20-bp target sequences (uppercase indicates a match with the consensus; lowercase indicates a mismatch; colored in red indicates the bulge base) and 3-bp PAM sequences (underlined) are shown.


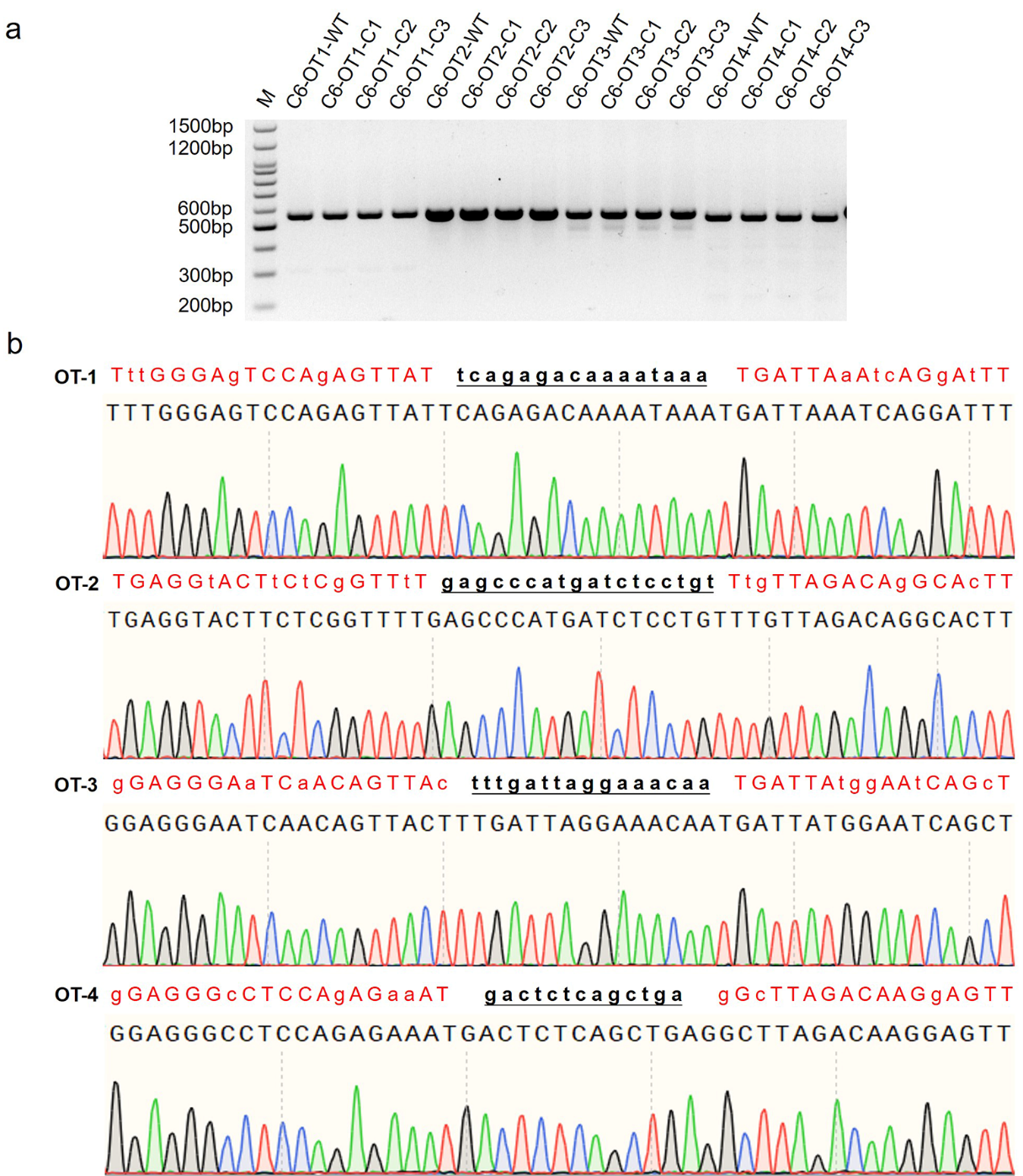


**Fig.S16 Off-target analysis of *CSN1* TALENs-C6 in the gene-replacement cows**

**(a)** T7EI cleavage analysis of the top 4 potential off-target sequences in the gene-replacement cows. OT-1-OT-4, top 4 potential off-target sites; M, 100-bp DNA ladder; WT, genome from WT cow; C1-C3, genome from the gene-replacement cows. **(b)** Representative chromatogram sequence analysis of the top 4 potential off-target sequences. The 20-bp target sequences (uppercase indicates a match with the consensus; lowercase indicates a mismatch; colored in red indicates the bulge base) and 3-bp PAM sequences (underlined) are shown.


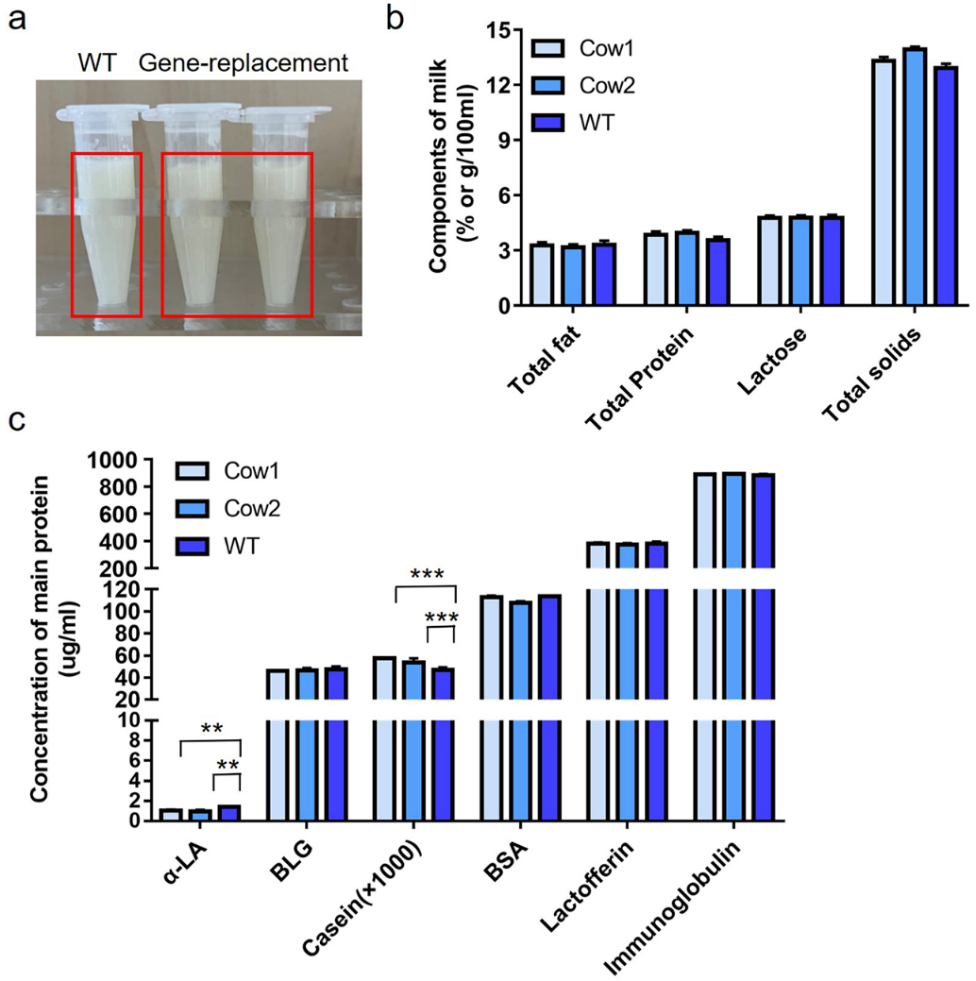


**Fig.S17 Analysis of milk composition**

**(a)** Milk samples from WT and site-specific gene replacement cows. **(b)** Total fat, total protein, lactose, and total solids in site-specific gene replacement and WT type cows’ milk. **(c)** The concentration of main protein in site-specific gene replacement and WT type cows’ milk. Cow1 and Cow2, site-specific gene replacement cows’ milk; WT, WT type cows’ milk. N=3, data are expressed as mean±SEM. ** P < 0.01; *** P < 0.001.
