# Supplementary material for: Versatile generation of precise gene edits in bovines using SEGCPN
Source: BMC Biol. 2023 Oct 20;21:226. doi: 10.1186/s12915-023-01677-0 (PMC10589966; doi:10.1186/s12915-023-01677-0)
Supplement: Supplementary file 2 — Additional file 2: Table S1. Summary of the PCR results of G418-resistant pMSTN-ESSEE (point mutation)-targeted clones. Table S2. Summary of the NT results of pMSTN-ESSEE (point mutation)-targeted BFFs. Table S3. List of the top 4 potential off-target effects of MSTN TALENs-M1. Table S4. Summary of the PCR results of G418-resistant pMSTN-ESSEE (11-bp deletion)-targeted clones. Table S5. Summary of the NT results of pMSTN-ESSEE (11-bp deletion)-targeted BFFs. Table S6. Summary of the PCR results of G418-resistant EGFP-targeted clones. Table S7. Summary of the nuclear transfer results of EGFP-targeted BFFs. Table S8. List of the top 4 potential off-target effects of SRY Cas9-sgRNA1. Table S9. Summary of the PCR results of G418-resistant gene-replacement clones. Table S10. Summary of the nuclear transfer results of gene-replacement BFFs. Table S11. List of the top 4 potential off-target effects of CSN1 TALENs-C3. Table S12. List of the top 4 potential off-target effects of CSN1 TALENs-C6. Table S13. Target sequences of the SRY sgRNAs used in this study. Table S14. TALEN recognition sequences and amino acid sequences of the repeat variable di-residues (RVDs) in the corresponding TALENs. Table S15. List of primers used in the present study. [file 12915_2023_1677_MOESM2_ESM.docx]

**Additional File2**

**Versatile generation of precise gene edits in bovines using SEGCPN**

Ming Wang^1,2,3^, Fangrong Ding^2^, Haiping Wang^2^, Ling Li^2^, Yunping Dai^2^^*^, ZhaoLin Sun^1,2,3*^,

Ning Li^2,3*^

^1^College of Animal Science and Technology, China Agricultural University, No.2 Yuanmingyuan Xilu, Beijing 100193, China.

^2^College of Biological Sciences, China Agricultural University, No.2 Yuanmingyuan Xilu, Beijing 100193, China.

^3^Beijing Capital Agribusiness Future Biotechnology Co., Ltd, No.75 Bingjiaokou Hutong, Beijing 100088, China.

^*^Correspondence and requests for materials should be addressed to Y.D. (email:[daiyunping@sina.com](mailto:daiyunping@sina.com)), Z. S. (email: [sunzhaolin@bjsnzz.com](mailto:sunzhaolin@bjsnzz.com)) or N.L. (lining@bjsngf.com.cn).

**TABLES-Additional File**

**Table S1 Summary of the PCR results of G418-resistant pMSTN-ESSEE (point mutation)-targeted clones**

| **Cell**  **line** | **Screening method** | **Cell colonies**  **analyzed by PCR** | **Positive**  **efficiency (%)** | **Homozygous positive efficiency (%)** | **Cell colonies selected for nuclear transfer** |
| --- | --- | --- | --- | --- | --- |
| 1401 | G418 | 48 | 64.6 (31/48) | 6.5 (2/31) | 1, 2, 9 |

**Table S2 Summary of the NT results of pMSTN-ESSEE (point mutation)-targeted BFFs**

| **No.of**  **cell clone** | **Gene**  **type** | **Re-construct embryos** | **Blastocysts**  **(%)** | **Recipients** | **Pregnancy at day 30** | **Fetus/Born bovine** |
| --- | --- | --- | --- | --- | --- | --- |
| #1 | heterozygous | 218 | 19.3 (42/218) | 2 | 1 | 0/0 |
| #2 | heterozygous | 267 | 19.5 (52/267) | 4 | 2 | 1/0 |
| #9 | homozygous | 280 | 17.1 (48/280) | 4 | 3 | 0/0 |

**Table S3 List of the top 4 potential off-target effects of *MSTN* TALENs-M1**

| Potential off-target Coord | TALEN recognition sequences | Left match | Right match | SP |
| --- | --- | --- | --- | --- |
|  | TAAGACCAAGTGAATGG **aggatgaatgagtatac** CTATCCCTCCAGGAGTCA | TAAGACCAAGTGAATGG | CTATCCCTCCAGGAGTCA | 17 |
| Chr3:37049451-37049502 | agAtACCAAGTGAATtG  **tccaaagtcatgcatc**  CcATCCCcCCAtGtGTCA | agAtACCAAGTGAATtG | CcATCCCcCCAtGtGTCA | 16 |
| Chr15:41102972-41103020 | gAAGAgCAAGTGAtTGG  **agtagtctgggaa** CTcTtCtTCCAGGAagCA | gAAGAgCAAGTGAtTGG | CTcTtCtTCCAGGAagCA | 13 |
| Chr19:86628-86673 | gAAtAtCAAGTGAggGG  **aatgagctca**  tTATCCCTCCAaGAaTCA | gAAtAtCAAGTGAggGG | tTATCCCTCCAaGAaTCA | 10 |
| Chr19:53841297-53841348 | TcAGACCAAGaGAATGc **agctgccggtccttgg**  CctTCCCTCCAGtgGcCA | TcAGACCAAGaGAATGc | CctTCCCTCCAGtgGcCA | 16 |

“Potential off-targets Coord” indicates the positions of the TALEN M1 hits in the cow genome assembly (chrom:start-end:strand). TALEN recognition sequences are the paired left and right recognition sequences and the spacer sequences between the left and right hits. Left match, the genomic sequence of the left hit (uppercase indicates a match with the consensus; lowercase indicates a mismatch). Right match, the genomic sequence of the right hit (uppercase indicates a match with the consensus; lowercase indicates a mismatch). SP, length of the spacer between the left and right hits.

**Table S4 Summary of the PCR results of G418-resistant pMSTN-ESSEE (11-bp deletion)-targeted clones**

| **Cell line** | **Screening method** | **Cell colonies**  **analyzed by PCR** | **Positive**  **efficiency (%)** | **Homozygous positive efficiency (%)** | **Cell colonies selected for nuclear transfer** |
| --- | --- | --- | --- | --- | --- |
| 1401 | G418 | 44 | 81.8 (36/44) | 2.8 (1/36) | 5, 6, 9 |

**Table S5 Summary of the NT results of pMSTN-ESSEE (11-bp deletion)-targeted BFFs**

| **No.of**  **cell clone** | **Gene**  **type** | **Re-construct embryos** | **Blastocysts**  **(%)** | **Recipients** | **Pregnancy at day 30** | **Fetus/Born bovine** |
| --- | --- | --- | --- | --- | --- | --- |
| #5 | heterozygous | 168 | 20.2 (34/168) | 2 | 1 | 0/0 |
| #6 | heterozygous | 201 | 21.4 (43/201) | 2 | 2 | 0/0 |
| #9 | homozygous | 292 | 18.5 (54/292) | 5 | 3 | 1/0 |

**Table S6 Summary of the PCR results of G418-resistant EGFP-targeted clones**

| **Cell line** | **Screening method** | **Cell colonies**  **analyzed by PCR** | **Positive**  **efficiency (%)** | **Cell colonies selected for nuclear transfer** |
| --- | --- | --- | --- | --- |
| 1401 | G418 | 29 | 72.4 (21/29) | 17, 20, 21 |
| 1004 | G418 | 12 | 58.3 (7/12) | 3, 4 |
|  | Total | 41 | 68.3 (28/41) |  |

**Table S7 Summary of the nuclear transfer results of EGFP-targeted BFFs**

| **No.of cell clone** | **Oocytes** | **Re-construct embryos** | **Blastocysts** | **Blastocysts** | **Recipients** | **Pregnancy at day 60** | **Live/Born bulls** |
| --- | --- | --- | --- | --- | --- | --- | --- |
| 1401 | 1905 | 1118 | 210 | 18.7% | 60 | 8 | 2/3 |
| 1004 | 1860 | 1079 | 313 | 29.0% | 20 | 4 | 1/1 |
|  | Total | 2197 | 523 | 23.8% | 80 | 12 | 3/4 |

**Table S8 List of the top 4 potential off-target effects of *SRY* Cas9-sgRNA1**

| Potential off-target Coord | sgRNA target sequences (20-bp) | PAM (3-bp) | Mismatches | Bulge Size |
| --- | --- | --- | --- | --- |
|  | CGCCTTTGTTAGCGAGAGTA | AGG |  |  |
| Chr28:40069605-40069628 | tGTCCTTTGTTAGaGAGAGTA | GGG | 2 | 1 |
| Chr28:40069608-40069630 | gtCCTTTGTTAGaGAGAGTA | GGG | 3 | 0 |
| Chr20:58892087-588922110 | aGTCCTTTGTTAGCaAGAGcA | AGG | 3 | 1 |
| Chr25:9092459-9092482 | CGCCTcccTTAGCGATGAGTA | AGG | 3 | 1 |

“Potential off-targets Coord” indicates the positions of the *SRY* Cas9-sgRNA1 hits in the cow genome assembly (chrom:start-end:strand). sgRNA target sequences, the 20-bp potential off-targets target sequences (uppercase indicates a match with the consensus; lowercase indicates a mismatch; colored in red indicates the bulge base). PAM, 3-bp protospacer adjacent motif sequences.

**Table S9 Summary of the PCR results of G418-resistant gene-replacement clones**

| **Cell line** | **Screening method** | **Cell colonies**  **analyzed by PCR** | **Positive**  **efficiency (%)** | **Cell colonies selected for nuclear transfer** |
| --- | --- | --- | --- | --- |
| 1001 | G418 | 34 | 61.8 (21/34) | 17, 20, 21 |
| 1003 | G418 | 64 | 54.7 (35/64) | 3, 4 |
|  | Total | 98 | 57.1 (56/98) |  |

**Table S10 Summary of the nuclear transfer results of** **gene-replacement BFFs**

| **No.of cell clone** | **Oocytes** | **Re-construct embryos** | **Blastocysts** | **Blastocysts** | **Recipients** | **Pregnancy at day 60** | **Live/Born cows** |
| --- | --- | --- | --- | --- | --- | --- | --- |
| 1001 | 1905 | 920 | 193 | 20.1% | 30 | 6 | 2/2 |
| 1003 | 1860 | 780 | 172 | 22.1% | 30 | 3 | 1/1 |
|  | Total | 1700 | 365 | 21.5% | 60 | 9 | 3/3 |

**Table S11 List of the top 4 potential off-target effects of *CSN1* TALENs-C3**

| Potential off-target Coord | TALEN recognition sequences | Left match | Right match | SP |
| --- | --- | --- | --- | --- |
|  | TCATCCTTACCTGTCTTGT **ggctgttgctcttgc** CAGGCCTGTGAGTACAGTA | TCATCCTTACCTGTCTTGT | CAGGCCTGTGAGTACAGTA | 15 |
| Chr1:74708380-74708434 | cCtTCCTTAtgTGTtTTGT  **ctttagtctcacacgc**  aAGcttTGTGAGTACAGTA | cCtTCCTTAtgTGTtTTGT | aAGcttTGTGAGTACAGTA | 16 |
| Chr6:99668039-99668100 | TtcTgtTTtCCTGTCTTGT  **gcagagactagatagctgtttaa** CAGGCtTGTaAGTACtGTt | TtcTgtTTtCCTGTCTTGT | CAGGCtTGTaAGTACtGTt | 23 |
| Chr15:68142740-68142807 | TCATCCTTcCCTcTCTacT  **cccaagcccctggcaactactcatcatag**  CAGagCTGTGgagACAGTA | TCATCCTTcCCTcTCTacT | CAGagCTGTGgagACAGTA | 29 |
| Chr28:12422791-12422842 | TCAaCtTTACCTGgCcTGT  **caggagctcagcc**  CAGtgCTGTGAGcACAGTg | TCAaCtTTACCTGgCcTGT | CAGtgCTGTGAGcACAGTg | 13 |

“Potential off-targets Coord” indicates the positions of the TALEN M1 hits in the cow genome assembly (chrom:start-end:strand). TALEN recognition sequences are the paired left and right recognition sequences and the spacer sequences between the left and right hits. Left match, the genomic sequence of the left hit (uppercase indicates a match with the consensus; lowercase indicates a mismatch). Right match, the genomic sequence of the right hit (uppercase indicates a match with the consensus; lowercase indicates a mismatch). SP, length of the spacer between the left and right hits.

**Table S12 List of the top 4 potential off-target effects of *CSN1* TALENs-C6**

| Potential off-target Coord | TALEN recognition sequences | Left match | Right match | SP |
| --- | --- | --- | --- | --- |
|  | TGAGGGACTCCACAGTTAT **ggtctttggtaagttgga** AACTGCTTGTCTAATCA | TGAGGGACTCCACAGTTAT | AACTGCTTGTCTAATCA | 18 |
| Chr11:99207125-99207177 | TttGGGAgTCCAgAGTTAT  **tcagagacaaaataaa** AAaTcCTgaTtTAATCA | TttGGGAgTCCAgAGTTAT | AAaTcCTgaTtTAATCA | 16 |
| Chr16:48648821-48648875 | TGAGGtACTtCtCgGTTtT  **gagcccatgatctcctgt**  AAgTGCcTGTCTAAcaA | TGAGGtACTtCtCgGTTtT | AAgTGCcTGTCTAAcaA | 18 |
| Chr23:14101821-14101873 | gGAGGGAaTCaACAGTTAc **tttgattaggaaacaa**  AgCTGaTTccaTAATCA | gGAGGGAaTCaACAGTTAc | AgCTGaTTccaTAATCA | 11 |
| Chr28:41241368-41241417 | gGAGGGcCTCCAgAGaaAT  **gactctcagctga**  AACTcCTTGTCTAAgCc | gGAGGGcCTCCAgAGaaAT | AACTcCTTGTCTAAgCc | 13 |

“Potential off-targets Coord” indicates the positions of the TALEN M1 hits in the cow genome assembly (chrom:start-end:strand). TALEN recognition sequences are the paired left and right recognition sequences and the spacer sequences between the left and right hits. Left match, the genomic sequence of the left hit (uppercase indicates a match with the consensus; lowercase indicates a mismatch). Right match, the genomic sequence of the right hit (uppercase indicates a match with the consensus; lowercase indicates a mismatch). SP, length of the spacer between the left and right hits.

**Table S13 Target sequences of the *SRY* sgRNAs used in this study**

| sgRNA | Target site (20-bp) | PAM (3-bp) |
| --- | --- | --- |
| sgRNA-1 | CGCCTTTGTTAGCGAGAGTA | AGG |
| sgRNA-2 | AAATAAGCACAAGAAAGTCC | AGG |
| sgRNA-3 | TTCCTTACTCTCGCTAACAA | AGG |

**Table S14 TALEN recognition sequences and amino acid sequences of the repeat variable di-residues (RVDs) in the corresponding TALENs**

| TALENs | TALEN recognition sequences (bold) and amino acid sequences of the RVDs |
| --- | --- |
| *MSTN*  Pair 1 | TAAGACCAAGTGAATGG aggatgaatgagtatac CTATCCCTCCAGGAGTCA  Left: **AAGACCAAGTGAATGG**  RVD: NI NI NN NI HD HD NI NI NN NG NN NI NI NG NN NN  Right: **GACTCCTGGAGGGATAG**  RVD: NN NI HD NG HD HD NG NN NN NI NN NN NN NI NG NI NN |
| *MSTN*  Pair 2 | TGTCCCTCTTCTTTCCTC cctgctcctttctcttct CTTCCCCCTCTCCCTTTA  Left: **GTCCCTCTTCTTTCCTC**  RVD: NN NG HD HD HD NG HD NG NG HD NG NG NG HD HD NG HD  Right: **AAAGGGAGAGGGGGAAG**  RVD: NI NI NI NN NN NN NI NN NI NN NN NN NN NN NI NI NN |
| *MSTN*  Pair 3 | TCTTCTTTCCTCCCTGCT cctttctcttctcttccc CCTCTCCCTTTACTGTCA  Left: **CTTCTTTCCTCCCTGCT**  RVD: HD NG NG HD NG NG NG HD HD NG HD HD HD NG NN HD NG  Right: **GACAGTAAAGGGAGAGG**  RVD: NN NI HD NI NN NG NI NI NI NN NN NN NI NN NI NN NN |
| *CSN1S1*  Pair 1 | TTGACAACCATGAAACTTC tcatccttacctgtctt GTGGCTGTTGCTCTTGCCA  Left: **TGACAACCATGAAACTTC**  RVD: NG NN NI HD NI NI HD HD NI NG NN NI NI NI HD NG NG HD  Right: **GGCAAGAGCAACAGCCAC**  RVD: NN NN HD NI NI NN NI NN HD NI NI HD NI NN HD HD NI HD |
| *CSN1S1*  Pair 2 | TGAAACTTCTCATCCTTAC ctgtcttgtggctgtt GCTCTTGCCAGGCCTGTGA  Left: **GAAACTTCTCATCCTTAC**  RVD: NN NI NI NI HD NG NG HD NG HD NI NG HD HD NG NG NI HD  Right: **CACAGGCCTGGCAAGAGC**  RVD: HD NI HD NI NN NN HD HD NG NN NN HD NI NI NN NI NN HD |
| *CSN1S1*  Pair 3 | TCATCCTTACCTGTCTTGT ggctgttgctcttgc CAGGCCTGTGAGTACAGTA  Left: **CATCCTTACCTGTCTTGT**  RVD: HD NI NG HD HD NG NG NI HD HD NG NN NG HD NG NG NN NG  Right: **ACTGTACTCACAGGCCTG**  RVD: NI HD NG NN NG NI HD NG HD NI HD NI NN NN HD HD NG NN |
| *CSN1S1*  Pair 4 | TCAAGTGAATTCTGAGGG actccacagttatg GTCTTTGGTAAGTTGGAA  Left: **CAAGTGAATTCTGAGGG**  RVD: HD NI NI NN NG NN NI NI NG NG HD NG NN NI NN NN NN  Right: **TCCAACTTACCAAAGAC**  RVD: NG HD HD NI NI HD NG NG NI HD HD NI NI NI NN NI HD |
| *CSN1S1*  Pair 5 | TTCTGAGGGACTCCACAGT tatggtctttggtaa GTTGGAAACTGCTTGTCTA  Left: **TCTGAGGGACTCCACAGT**  RVD: NG HD NG NN NI NN NN NN NI HD NG HD HD NI HD NI NN NG  Right: **AGACAAGCAGTTTCCAAC**  RVD: NI NN NI HD NI NI NN HD NI NN NG NG NG HD HD NI NI HD |
| *CSN1S1*  Pair 6 | TGAGGGACTCCACAGTTAT ggtctttggtaagttgga AACTGCTTGTCTAATCA  Left: **GAGGGACTCCACAGTTAT**  RVD: NN NI NN NN NN NI HD NG HD HD NI HD NI NN NG NG NI NG  Right: **GATTAGACAAGCAGTT**  RVD: NN NI NG NG NI NN NI HD NI NI NN HD NI NN NG NG |

**Table S15 List of primers used in the present study**

| Primers | Sequence |
| --- | --- |
| EGFP-F | TATATCATGGCCGACAAGCA |
| EGFP-R | ATACTTTCTCGGCAGGAGCA |
| mOCT4-F | AGGGGTGAGAGGACCTTGA |
| mOCT4-R | GCGATCCCTGAACATGTCCAT |
| MSTN-F | AGTGAAAGAATAAAGGGAATA |
| MSTN-R | AGTTAGAGGGTAACGACAG |
| P1 | CACCATATTTAATCAATAAG |
| P2 | GCCGAGAAAGTATCCA |
| P3 | ACCTAAAATTTCTAAACCAAA |
| P4 | AGGATTAGCAAATTGTAG |
| P5 | TGAACCTCTGGGGTTTGCT |
| SRY-F | TGTGCCAAGACCACATACTC |
| SRY-R | AGAAAGACCAAAGAACAGACC |
| P6 | TCCTCCTATTTTATTACATG |
| P7 | CCTGGACGTAGCCTTC |
| P8 | TGCTCGACGTTGTCACT |
| P9 | GGAATGCATAAATGTCTGATT |
| P10 | CGGCAACTACAAGACC |
| P11 | ACAGTCTGTGAAGTTACC |
| P12 | CCCGGGCTATAAATATCGAC CCCGGGCTATAAATATCGAC |
| P13 | CCTTCAGCTCGATGCGGTTCA |
| CSN1-E1-F | AGAGCAAAATTAAAAACTAAA |
| CSN1-E1-R | GGATGGAGGAGCCTGGCAG |
| CSN1-E1-R’ | AGGACCAAATTATTAGATCTT |
| CSN1-E18-F | TGCCTTTCTTTTGATTATATC |
| CSN1-E18-R | CTAGAAAGGGAACATACACAT |
| P14 | TGCCAGTTAATTCTAGGAGTA |
| P15 | CGAGATTACACCATTGCAC |
| P16 | CTCCTGCCGAGAAAGTATC |
| P17 | ATCTACAGAAAACGTGACTTT |
| P18 | CCCTACAATTTTCTTGGATAT |
| NEO-F | GGCAGGTAGCCGGATCAAGCG |
| NEO-R | CAAGATGGATTGCACGCAGGTTCTC |
| GAPDH-F | GCAAGTTCCACGGCACAG |
| GAPDH-R | CGCCAGTAGAAGCAGGGAT |
| EGFP-DIG-F | ATGGTGAGCAAGGGCGAGGAG |
| EGFP-DIG-R | TTACTTGTACAGCTCGTCCATGC |
| HLA-DIG-F | GCAATCCCTATCAAGCTACCA |
| HLA-DIG-R | ATCCTGCTGTGATGTATGTCG |
| M1-OT1-F | CTGATGCCATTCCAGTTCCT |
| M1-OT1-R | TGGAAAATGTTGGATCATTCACT |
| M1-OT2-F | CAGCTGCTATGTGCCAGGTA |
| M1-OT2-R | CCATTCATCCATCCTGCTCT |
| M1-OT3-F | CTTGCAACAGCAAACTTGGA |
| M1-OT3-R | AGGGATCAAACCCAGGTCTC |
| M1-OT4-F | TAGGCCCCTGATGAAGTCAC |
| M1-OT4-R | GTCTCCAGCCTCCTCTCCTT |
| sgRNA-OT1-F | AGTTTCGGCCTCACTGACAC |
| sgRNA-OT1-R | CTTGTATTGGCAGGCAGGTT |
| sgRNA-OT2-F | AGTTTCGGCCTCACTGACAC |
| sgRNA-OT2-R | CTTGTATTGGCAGGCAGGTT |
| sgRNA-OT3-F | AGCATTCAGATGTGGGACCT |
| sgRNA-OT3-R | TGGATTCCAGAAAACCTTCG |
| sgRNA-OT4-F | CGCACAGAACTTGGAAACCT |
| sgRNA-OT4-R | GAGCACTGGGAGGTCAGTTC |
| C3-OT1-F | AGGTCCCAGTGGCTTAAATG |
| C3-OT1-R | AAGGAAGGGATCATGGGAAC |
| C3-OT2-F | CAGGTGGCACAGTGGTAAAG |
| C3-OT2-R | TTCTTAAGCGGCTGTGGTCT |
| C3-OT3-F | CTTACCCCAGCACACAGCTT |
| C3-OT3-R | GGAGAATCCCCTGGAGTAGG |
| C3-OT4-F | GTTGCCATTTCCTTTTCCAA |
| C3-OT4-R | GCATTCCCATTTTCCATGTC |
| C6-OT1-F | AACAGCTGCGGTTCAGAAAT |
| C6-OT1-R | TCCAGGCAACCACTTTGTATC |
| C6-OT2-F | CTTTGTGACCCCGTGGACT |
| C6-OT2-R | CTGGAGCCCAGAAATCTGAA |
| C6-OT3-F | CCAAGTGTCAGCCATCTGAA |
| C6-OT3-R | CCTTCTGGTCCTGAGGTCAC |
| C6-OT4-F | GACTCACAGGAAGGCAGAGC |
| C6-OT4-R | CACAGACACGCTTCACCACT |
